# Regulatory T cells and peripheral immune tolerance: key mechanisms and therapeutic strategies for cardiovascular diseases

**DOI:** 10.3389/fimmu.2026.1776040

**Published:** 2026-03-26

**Authors:** Mengna Lin, Wenbo Li, Yawen Wu, Miao Li, Yongnan Li, Yinglu Zhao

**Affiliations:** 1 Department of Cardiac Surgery, Lanzhou University Second Hospital, Lanzhou University, Lanzhou, China; 2 Second Clinical Medical College, Lanzhou University, Lanzhou, China; 3 Department of Cardiology, Lanzhou University Second Hospital, Lanzhou University, Lanzhou, China

**Keywords:** cardiovascular diseases, immune inflammation, molecularmechanisms, regulatory T cells, treatment

## Abstract

Cardiovascular diseases (CVDs) remain the leading global cause of disability and mortality, with chronic immune inflammation as their central pathological mechanism. Regulatory T cells (Tregs) and the peripheral immune tolerance they mediate hold a pivotal position and therapeutic potential in CVDs. This review provides a comprehensive overview of Tregs biology, their dysfunction in major CVDs, current therapeutic strategies targeting Tregs, and future directions for achieving immune homeostasis. By moving beyond the traditional focus on numerical deficiency, we highlight the critical role of functional heterogeneity and cellular dysfunction in CVDs pathogenesis, and discuss the emerging therapeutic concept of moving from delaying disease progression to restoring immune homeostasis.

## 1 Introduction

The cardiovascular system, comprising the heart and blood vessels (arteries, veins, capillaries), gives rise to various diseases when structurally or functionally impaired. Clinically, CVDs primarily encompass four categories: coronary artery disease, cerebrovascular disease, peripheral artery disease, and aortic atherosclerosis (1). Recent evidence confirms chronic immune inflammation as the central driver throughout CVDs initiation and progression (2, 3). T cell-mediated adaptive immunity has thus become a focal point for exploring CVDs immunopathology and novel therapies (4, 5). Among infiltrating T cells, Tregs play a critical role in maintaining homeostasis, immune balance, and tolerance (6, 7). The discovery of regulatory T cells and the elucidation of their FOXP3 function represent a breakthrough in the field of immunology. The 2025 Nobel Prize in Physiology or Medicine was awarded for groundbreaking discoveries concerning peripheral immune tolerance. This revealed that immune defense relies not only on the “offensive” response of effector T cells but also equally on Tregs-mediated “inhibitory” regulation to prevent autoimmunity and maintain internal stability. This mechanistic insight provides a strong rationale for Tregs-targeted therapy in CVDs, offering the potential to move from symptom management towards precise intervention and from macroscopic treatment towards precise microscopic immunomodulation. This review explores this concept, focusing on the goal of actively restoring immune homeostasis and potentially reversing cardiovascular pathology. This review advances the paradigm from merely delaying disease progression to actively restoring immune homeostasis and reversing cardiovascular pathology. While animal models—particularly genetically modified mice—have provided invaluable mechanistic insights into Tregs biology and cardiovascular immunopathology, inherent species differences, controlled experimental conditions, and the absence of comorbiditiy burden limit direct extrapolation to human disease.

## 2 Tregs

A profound understanding of Tregs function in CVDs requires clarity on their fundamental biology, including their discovery, developmental origins, core suppressive mechanisms, and their cornerstone role in maintaining immune homeostasis. Examining how Tregs dysfunction disrupts immune tolerance to drive CVDs pathogenesis then establishes the theoretical basis for their therapeutic targeting.

### 2.1 Discovery and research progress of Tregs

The “suppressor T cell” hypothesis, first proposed by Gershon and Kondo in 1970, revealed a T cell subset capable of negatively regulating immune responses (8). Subsequent work in neonatally thymectomized and bone marrow-reconstituted mice showed that both antibody responses and their suppression depended on thymus-derived cells, prompting the systematic identification of anti-inflammatory thymic populations (9).

In 1995, Sakaguchi’s team demonstrated that depleting or reducing CD4^+^CD25^+^ T cells in mice induced autoimmune disease, while their transfer prevented it. This work first identified and isolated a distinct immunosuppressive T cell population via surface markers, also showing that transferring CD4^+^CD25^-^ T cells to immunodeficient mice enhanced allogeneic skin graft rejection, which was normalized by co-transfer of CD4^+^CD25^+^ T cells (10). These CD4^+^CD25^+^ T cells were established as crucial for establishing and maintaining peripheral self-tolerance, defining Tregs as a distinct functional subset (11, 12). Later studies confirmed Tregs as a lineage functionally and developmentally distinct from other T cells, central to maintaining immune self-tolerance (13).

The transcription factor FOXP3 is the lineage-defining regulator of Tregs, governing their development, differentiation, and expression of immunosuppressive genes throughout their lifecycle. *FOXP3* is primarily and specifically expressed in thymic and peripheral natural Tregs, serving as a more specific and reliable marker than CD25 or CTLA-4 for distinguishing Tregs from activated/effector/memory T cells (14, 15), making CD4^+^CD25^+^FOXP3^+^ the most widely accepted Tregs signature (16). Genetically, FOXP3 deficiency causes Tregs loss and IPEX syndrome, confirming the axiom “no FOXP3, no Tregs function, “ a phenotype rescuable by functional Tregs transfer (17, 18). Functionally, FOXP3 induces immunosuppressive programs involving molecules like IL-10 and modulates Tregs suppressive activity by integrating TCR signaling. Crucially, ectopic *FOXP3* expression in peripheral non-regulatory CD4^+^CD25^-^ T cells confers full suppressive capacity, driving their differentiation into functional Tregs and confirming its deterministic role (19, 20). Collectively, these findings establish FOXP3 as a central regulator of Tregs biology. The generation of Tregs via FOXP3 transduction in naive T cells is therefore under investigation as a potential strategy for treating autoimmune and inflammatory diseases, as well as for inducing transplant tolerance.

### 2.2 Origin of Tregs

Tregs develop primarily via two complementary pathways: thymus-derived natural Tregs (nTregs/tTregs) and peripherally induced Tregs (pTregs), which differ in origin, inducing microenvironment, and functional emphasis. tTregs mature in the thymus, where TCR interaction with self-peptide-MHC complexes (class I or II) induces FOXP3 expression, mediating dominant tolerance to control T cell self-reactivity and maintain systemic immune homeostasis (21, 22). pTregs derive from peripheral naive CD4^+^ T cells induced by specific microenvironmental cues like TGF-β, IL-2, and retinoic acid (23–25); they possess a broader TCR repertoire and primarily mediate local immunoregulation and tolerance to foreign antigens. Antigen-presenting cells (APCs), particularly dendritic cells, are also critical for this conversion (26, 27). Furthermore, *in vitro* generated induced Tregs (iTregs) constitute a third source, enabling adoptive Tregs transfer therapy for CVDs by expanding numbers or generating functionally optimized iTregs, thereby extending the scope of adaptive tolerance, as detailed in the therapy section.

### 2.3 Immunosuppressive mechanisms of Tregs

**Figure 1:**
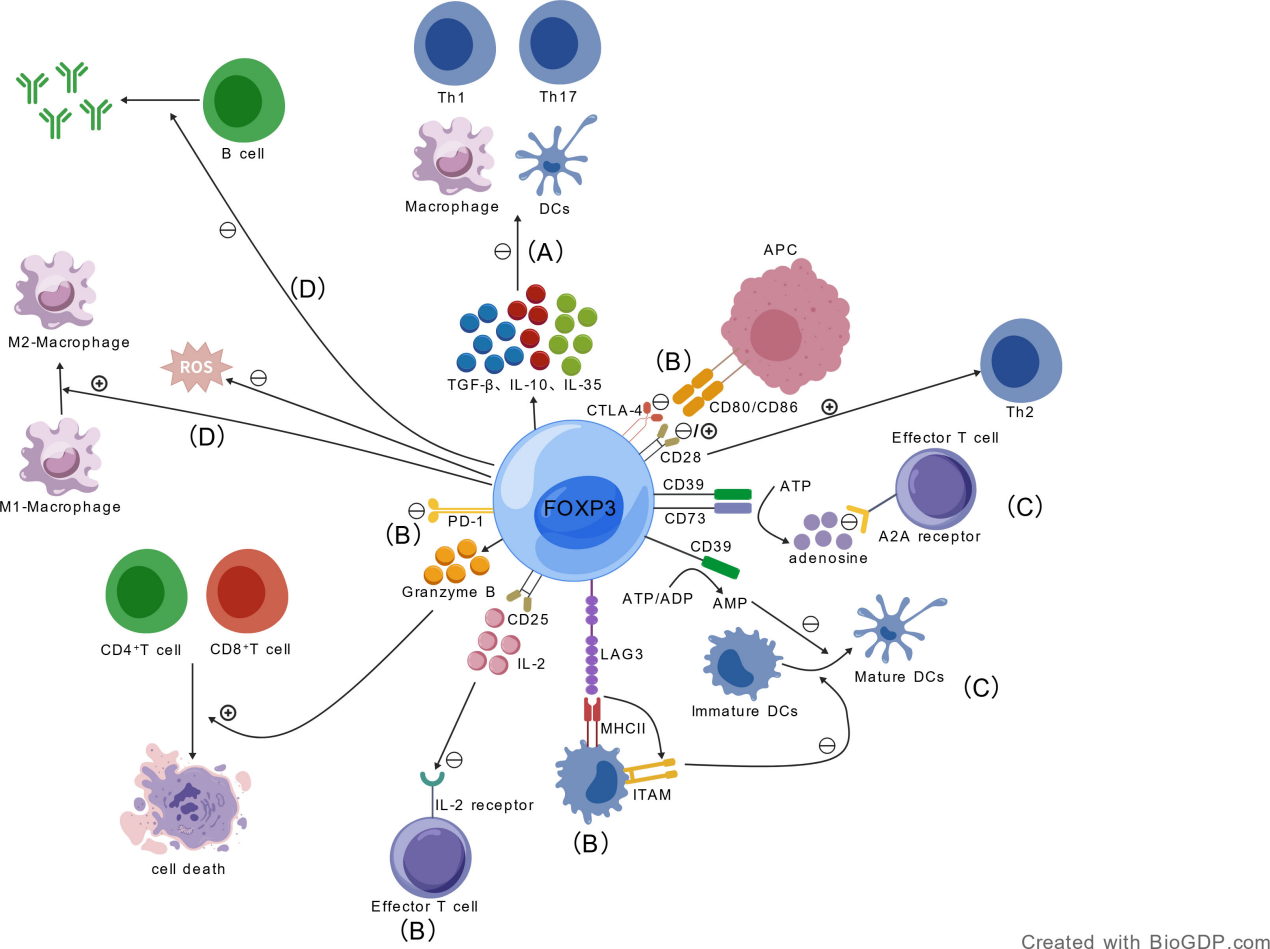
Multifaceted immunosuppressive mechanisms of FOXP3^+^ regulatory T cells (Tregs). FOXP3, the master transcription factor of Tregs, orchestrates diverse immunosuppressive pathways to modulate immune homeostasis and inflammation: **(A)** Cytokine-mediated inhibition: Secretion of TGF-β, IL-10, and IL-35 to suppress activation of effector T cells (Th1/Th17), antigen-presenting cells (APCs, e.g., macrophages, dendritic cells), and pro-inflammatory factor release; **(B)** Immune checkpoint and receptor-mediated regulation: Competitive IL-2 consumption via CD25; CTLA-4-mediated CD80/CD86 trans-endocytosis on APCs; PD-1/LAG3-dependent inhibitory signaling to balance T cell activation; **(C)** Metabolic modulation: CD39/CD73-catalyzed ATP-to-adenosine conversion to suppress effector T cell function; regulation of the Tregs-cytokine-sortilin1 immunometabolic axis via IFN-γ/JAK/STAT pathway; **(D)** Cellular phenotype and functional regulation: Promotion of M1-to-M2 macrophage polarization; granzyme B-dependent killing of effector T cells; suppression of B cell antibody production; and modulation of endothelial function, lipid metabolism, and extracellular matrix remodeling. APC, antigen-presenting cell; DC, dendritic cell; FOXP3, forkhead box P3; IFN-γ, interferon-γ; IL, interleukin; LAG3, lymphocyte activation gene 3; PD-1, programmed cell death protein 1; ROS, reactive oxygen species; TGF-β, transforming growth factor-β.

Tregs execute immunosuppressive functions through multiple mechanisms and targets **Figure 1**. Tregs have precise intracellular signaling pathways, transcriptional regulation, and epigenetic modifications that underlie their functional specificity and stability.

#### 2.3.1 Cytokines TGF-β, IL-10, and IL-35

Tregs secrete inhibitory cytokines such as TGF-β, IL-10, and IL-35, which directly suppress the activation and function of antigen-presenting cells (e.g., macrophages, dendritic cells), effector T cells (e.g., Th1, Th17), and other immune cells (28–30), while also reducing pro-inflammatory factor release to alleviate vascular wall inflammation (31). TGF-β binds to TβRI/TβRII on target cells, triggering SMAD2/3 phosphorylation. The SMAD2/3-SMAD4 complex translocates to the nucleus, suppressing pro-inflammatory gene transcription (e.g., IL-6, IFN-γ).Autocrine TGF-β enhances FOXP3 transcription via the SMAD pathway in Tregs, stabilizing their immunosuppressive phenotype (32). IL-10 engages IL-10R to activate JAK1/TYK2-STAT3 signaling, inhibiting NF-κB/AP-1 pathways and reducing TNF-α/IL-1β release (33). In Tregs, IL-10 upregulates CTLA-4/CD25 via STAT3, forming an immunosuppressive feedback loop (34). IL-35 forms a p35/EBI3 heterodimer, binds IL-12Rβ2/gp130, and activates STAT1/4 to suppress Th1/Th17 differentiation and promote Tregs proliferation (35).

#### 2.3.2 IL-2 receptor (CD25)

Tregs competitively consume IL-2 in the microenvironment by highly expressing the IL-2 receptor (CD25), thereby depriving effector T cells of this essential survival and proliferation signal (36, 37). IL-2 binding to CD25 activates JAK1/JAK3-STAT5 signaling: phosphorylated STAT5 binds FOXP3’s CNS2 region, recruiting p300 to enhance FOXP3 transcription, while upregulating Bcl-2 and CTLA-4 to maintain Tregs survival and function (38, 39).

#### 2.3.3 CTLA-4

Through contact-dependent mechanisms, Tregs use Cytotoxic T-Lymphocyte Antigen-4 (CTLA-4) to bind CD80/CD86 on antigen-presenting cells, inducing their trans-endocytosis and downregulation, which subsequently inhibits T cell activation and differentiation (40–42). CTLA-4-mediated immunosuppression is achieved through the following mechanisms: Extracellularly, Tregs bind to CD80/CD86 on APCs via CTLA-4, capturing and internalizing these costimulatory molecules through trans-endocytosis, leading to their degradation within Tregs, thereby depriving effector T cells of CD28 costimulatory signals (43). In Tregs, CTLA-4 signaling, mediated by Foxp3 expression, inhibits the PI3K-Akt-mTOR pathway, reducing glycolysis and indirectly supporting oxidative phosphorylation to sustain Tregs suppressive function (44). In APCs, CTLA-4 engagement can activate the PI3K/Akt/mTOR axis, promoting FoxO1 nuclear exclusion, reducing the transcription of autophagy-related genes, and diminishing APC immunogenicity (45).

#### 2.3.4 CD28

CD28 serves as the core co-stimulatory molecule for naive T cell activation, exhibiting dual functions: it amplifies immune responses by promoting cytokine secretion, proliferation, and survival, while also inducing CTLA-4 expression to inhibit excessive activation. Furthermore, CD28 drives Th2 cell differentiation—which exerts protective effects in autoimmunity—and is essential for Tregs homeostasis (46). CD28 not only activates FOXP3 transcription in naive CD4^+^CD25^-^ T cells through its own induced NF-κB-dependent signaling pathway. It also drives the nuclear translocation of RelA/NF-κB, but not c-Rel. This allows RelA dimers to directly bind to a novel κB site on the FOXP3 gene. Consequently, it initiates FOXP3 transcription by mediating histone acetylation and recruiting RNA polymerase II. Simultaneously, it regulates the binding of NFAT to the FOXP3 promoter to precisely control transcriptional efficiency. Through these mechanisms, it ultimately regulates Tregs differentiation and peripheral immune tolerance (47).Superagonistic anti-CD28 antibodies preferentially expand and potently activate natural CD4^+^CD25^+^CTLA-4^+^FOXP3^+^ Tregs *in vivo*, effectively preventing or alleviating autoimmune disease symptoms and inducing remission (48).

#### 2.3.5 PD-1

Programmed cell death protein 1 (PD-1), a key immunosuppressive molecule on immune cells, balances immune activation by initiating inhibitory signaling upon ligand binding: phosphorylating ITSM and ITIM motifs to recruit SHP-2/SHP-1, leading to dephosphorylation of TCR-associated CD3ζ and ZAP70, thereby suppressing the PI3K/Akt/Ras pathway and CD8^+^ T cell activation, proliferation, and IL-2 production (49). In chronic viral infection models, Tregs expansion and PD-1 upregulation suppress CD8^+^ T cell proliferation and IFN-γ secretion through this mechanism (50). Conversely, PD-1 expression on Tregs can limit their activation and function via the PI3K-AKT pathway to modulate tolerance, while simultaneously increasing IFN-γ production (49, 51).

These seemingly contradictory findings suggest that PD-1 on Tregs mediates context-dependent bidirectional immunoregulation via PD-L1/PD-L2 binding, rather than simply suppressing immune cell activation. Under homeostatic conditions, PD-1 constrains Tregs overactivation via PI3K-AKT to maintain balanced suppression. In pathological settings like chronic infection, inflammatory signals expand Tregs and enhance their baseline suppression: PD-1 both moderately restrains Tregs and directly inhibits CD8^+^ T cells via PD-L1 binding, while compensatory signals in the microenvironment override PD-1-mediated self-inhibition, sustaining a potent immunosuppressive phenotype. The detailed molecular mechanisms, functional heterogeneity across microenvironments, and signaling crosstalk networks require further systematic investigation.

#### 2.3.6 CD39and CD73

Tregs also express CD39 and CD73 to catalyze extracellular ATP conversion to adenosine, which suppresses effector T cell activity via the A2A receptor, thereby limiting inflammation (52–54). The ectoenzyme CD39 hydrolyzes ATP/ADP to AMP, enhancing Tregs-mediated suppression of ATP-driven dendritic cell maturation (31). CD73 converts AMP to adenosine, activating the cAMP-PKA pathway in target cells to inhibit pro-inflammatory gene transcription (55).CD39 can also inhibit the activation of the NLRP3 inflammasome. This inhibition subsequently attenuates lipopolysaccharide-induced inflammation, apoptosis, and oxidative stress injury in renal tubular epithelial cells. Consequently, it exerts a protective effect against acute kidney injury caused by sepsis (56).

#### 2.3.7 LAG3

Tregs-expressed Lymphocyte Activation Gene 3 (LAG3) exhibits high affinity for MHC class II molecules. LAG3 binding to MHCII activates an ITAM-mediated inhibitory pathway, suppressing dendritic cell maturation and their ability to stimulate T cells (57). Specifically in Tregs, LAG3 restricts Myc expression and glycolytic metabolism by regulating the PI3K-Akt-Rictor pathway. This mechanism is crucial for maintaining the suppressive function of Tregs (58). In dendritic cells (DCs), LAG3 signaling limits their T cell priming capacity. It achieves this by inhibiting the glycolytic metabolism and the expression of co-stimulatory molecules, such as CD86 and CD40, in DCs. Consequently, LAG3-deficient DCs exhibit higher CD86 expression, enhanced glycolytic activity, and an improved capacity to drive T cell effector differentiation (59).

#### 2.3.8 Gut microenvironment and metabolism

Recent studies have revealed close interactions between Tregs, metabolism, and the gut microenvironment. High-fat diet-induced gut dysbiosis disrupts the intestinal immune barrier, significantly reducing Tregs proportions and increasing pro-inflammatory Th17 cells in the lamina propria, thereby disrupting Th17/Tregs balance and exacerbating systemic inflammation (60, 61). Short-chain fatty acids (SCFAs) (e.g., butyrate) from gut microbiota bind Tregs’GPR41/GPR43, activating AMPK to enhance FOXP3 expression and maintain Th17/Tregs balance (62).

Sortilin-1, a VPS10p domain receptor, is genetically linked to cardiovascular diseases through genome-wide association studies. Tregs suppress hepatocyte IFN-γ release, a key inflammatory mediator regulating sortilin-1. Tregs deficiency elevates hepatic IFN-γ, activating the JAK/STAT pathway to induce STAT1 phosphorylation; p-STAT1 then binds the SORT1 gene regulatory region to suppress sortilin-1 transcription and expression. As a critical lipoprotein metabolism receptor, stable sortilin-1 expression maintains metabolic balance and modulates inflammation, establishing a Tregs-cytokine-sortilin1 immunometabolic axis (63).

#### 2.3.9 Other immunosuppressive mechanisms

Tregs also promote macrophage polarization from pro-inflammatory M1 to anti-inflammatory M2 phenotypes, enhance efferocytosis, and inhibit inflammatory cytokine and ROS production (28, 64). Furthermore, Tregs reduce inflammatory cell infiltration via granzyme B-dependent, perforin-independent killing of T cells (65). Tregs also suppress B cell antibody production and class-switch recombination, improve endothelial function, regulate lipid metabolism, inhibit macrophage-to-foam-cell transformation, increase plaque smooth muscle cell and collagen content, and prevent abnormal cardiovascular matrix remodeling and fibrosis. These functions will be detailed in subsequent disease-specific sections.

## 3 Cardiovascular diseases and tregs

The balance between Tregs function and other CD4^+^ T cell subsets like Th17 is crucial in CVDs immunopathophysiology (66). Enhancing Tregs function or quantity effectively slows disease progression (42); exogenous Tregs adoptive transfer or endogenous Tregs expansion successfully inhibits progression in multiple cardiovascular diseases (67).

**Figure 2:**
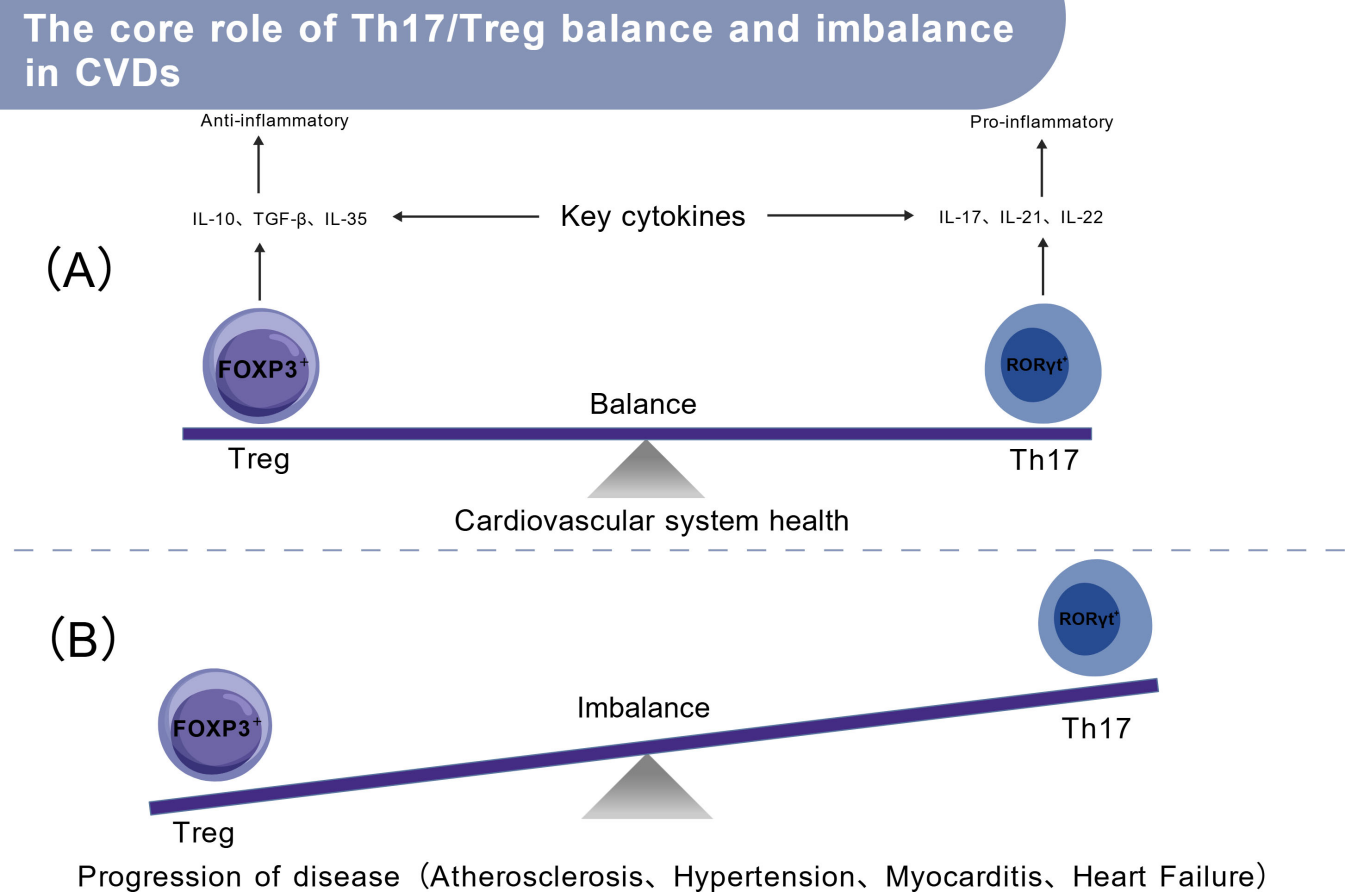
The core role of Th17/Treg balance and imbalance in cardiovascular diseases (CVDs). **(A)** Balanced state: FOXP3+ Tregs secrete anti-inflammatory cytokines (IL-10, TGF-β, IL-35), while Th17 cells produce pro-inflammatory cytokines (IL-17, IL-21, IL-22); the equilibrium of these two subsets maintains cardiovascular homeostasis; **(B)** Imbalanced state: Disrupted FOXP3 expression leads to Th17/Treg imbalance, promoting the progression of CVDs including atherosclerosis, hypertension, myocarditis, and heart failure. Abbreviations: CVDs, cardiovascular disease; IL, interleukin; TGF-β, transforming growth factor-β; Treg, regulatory T cell.

The differentiation fate of naive CD4^+^ T cells dictates immune outcomes, with specific cytokines driving polarization toward pro-inflammatory Th17 or anti-inflammatory Tregs (66). In heart failure and hypertension models, Th17 cells upregulate LOX and MMPs via the IL-17/ERK1/2-AP-1 pathway, driving myocardial fibrosis, inflammation, and adverse cardiac remodeling. Tregs counteract this through the IL-10/JAK1-STAT3 pathway, suppressing inflammation, promoting tissue repair, and maintaining immune tolerance (68). In atherosclerosis, Th17 and Th1 cells drive plaque inflammation, while Tregs exert anti-inflammatory and plaque-stabilizing effects (69). Thus, the dynamic Th17/Tregs balance underpins cardiovascular immune homeostasis (**Figure 2**).

Clinically, disrupted Th17/Tregs balance is a common feature of CVDs. Elevated Th17 and reduced Tregs proportions are observed in atherosclerosis (69), heart failure (70), rheumatic heart disease (71), and resistant hypertension (72). However, a deep understanding of Tregs protective mechanisms requires analysis within specific disease contexts. The following sections will detail Tregs functional states and mechanisms across specific cardiovascular diseases.

### 3.1 Atherosclerosis

CD4^+^CD25^+^FOXP3^+^ Tregs are crucial for maintaining immune homeostasis, and their functional impairment is closely linked to the initiation and progression of atherosclerosis (73, 74). In animal models like ApoE^-^/^-^ mice, a decreased peripheral Tregs ratio correlates with aggravated atherosclerotic lesions and enhanced plaque instability (73, 75). Clinical studies confirm low numbers of FOXP3+ Tregs in human atherosclerotic plaques and a reduced circulating proportion of Tregs, which is associated with impaired cellular function in patients with acute coronary syndrome (76, 77). These findings indicate that Tregs deficiency is not merely a biomarker but directly contributes to atherogenesis.

**Figure 3:**
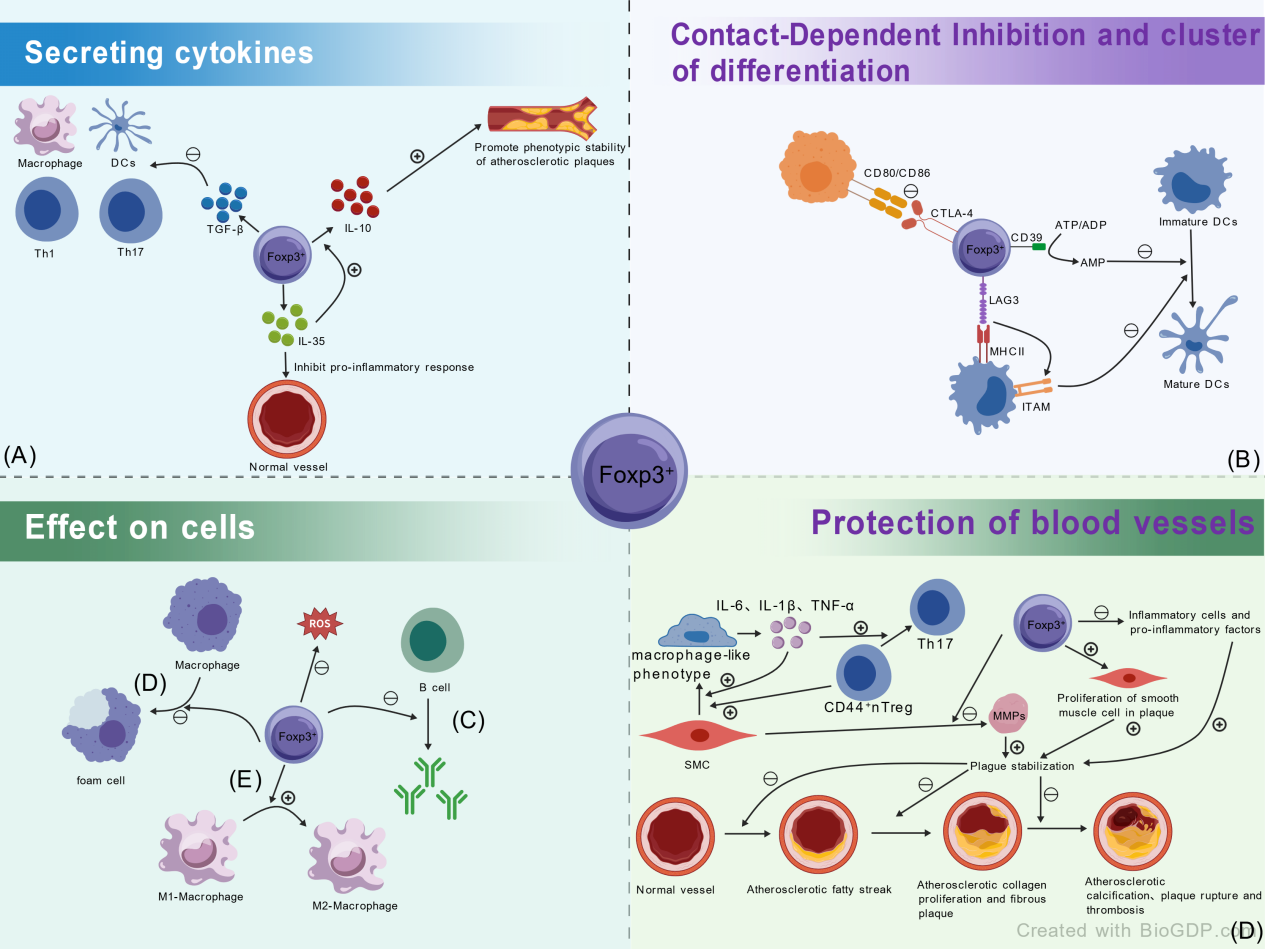
Multi-level and multi-target anti-atherogenic mechanisms of Tregs. Tregs exert anti-atherogenic effects through diverse pathways: **(A)** Cytokine and CTLA-4-mediated regulation: Secretion of TGF-β, IL-10, IL-35 to suppress immune cell activation and inflammation; CTLA-4 binds and trans-endocytoses APCs’ CD80/CD86 to inhibit T cell activation; **(B)** Ectoenzyme and receptor function: CD39 hydrolyzes ATP/ADP to AMP, suppressing DC maturation; LAG3 binds MHC class II to inhibit DC and T cell activity; **(C)** B cell modulation: Directly suppresses B cell antibody production and class-switch recombination, attenuating pro-atherogenic effects; **(D)** Vascular protection: Improves endothelial function, regulates lipid metabolism, inhibits macrophage foam cell formation, and enhances plaque stability by reducing inflammation and fibrosis; **(E)** Macrophage polarization and novel signaling: Promotes M1-to-M2 macrophage polarization and efferocytosis. APC, antigen-presenting cell; AS, atherosclerosis; DC, dendritic cell; IL, interleukin; LAG3, lymphocyte activation gene 3; MMP, matrix metalloproteinase; ROS, reactive oxygen species; SMC, smooth muscle cell; TGF-β, transforming growth factor-β.

Tregs exert anti-atherogenic effects through multi-level, multi-target mechanisms, primarily including: First, secretion of inhibitory cytokines TGF-β, IL-10, and IL-35. TGF-β suppresses the activation and function of APCs (e.g., macrophages, DCs), effector T cells (Th1, Th17), and other immune cells to delay progression (28–30). IL-10 deficiency accelerates lesions and destabilizes plaques, while its overexpression inhibits disease; it is key for stabilizing established plaques (78). IL-35, which suppresses pro-inflammation and promotes IL-10 secretion, represents a potential therapeutic target (79). Concurrently, Tregs use CTLA-4 to bind and trans-endocytose CD80/CD86 on APCs, downregulating these co-stimulatory molecules to inhibit APC and T cell activation/differentiation (40–42). Second, the ectoenzyme CD39, expressed by Tregs, hydrolyzes ATP/ADP to AMP, enhancing suppression of ATP-driven dendritic cell maturation (31). Tregs-expressed LAG3 has high affinity for MHC class II; its binding activates an ITAM-mediated inhibitory pathway, suppressing DC maturation and T cell stimulatory capacity (57).Third, Tregs also protect against AS by modulating B cell activity. They directly suppress B cell antibody production and class-switch recombination, curbing their pro-atherogenic role (80, 81). Fourth, at the vascular level, Tregs improve endothelial function, regulate lipid metabolism, and inhibit macrophage-to-foam-cell transformation. Tregs adoptive transfer in murine models reduces inflammatory cell infiltration and pro-inflammatory cytokine secretion, while increasing plaque smooth muscle cell and collagen content to enhance stability. They also inhibit SMC secretion of MMPs, preventing aberrant cardiovascular matrix remodeling and fibrosis (73, 75).Fifth, Tregs promote macrophage polarization from pro-inflammatory M1 to anti-inflammatory M2 phenotypes, enhance efferocytosis, limit plaque progression and necrotic core formation, and inhibit inflammatory cytokine and ROS production (28, 64). Notably, recent advances show that amyloid precursor protein on endothelial cells interacts with macrophage CD74 to generate migrasomes. Blocking CD74 disrupts migrasome-mediated signaling and attenuates AS progression, suggesting the migrasome-APP-CD74 axis as a novel therapeutic target for vascular inflammation (82) **Figure 3**.

Furthermore, macrophage-like vascular smooth muscle cells (VSMCs) secrete inflammatory factors (IL-6, IL-1β, TNF-α) that promote VSMCs phenotypic switching, destabilize tTregs, and drive their differentiation into pathogenic Th17 cells, exacerbating vasculitis. Conversely, CD44^+^ tTregs actively reprogram VSMCs toward a macrophage-like phenotype, creating a vicious cycle (83).

During atherosclerotic regression, FOXP3^+^ Tregs numbers increase significantly. They actively resolve plaque inflammation and promote tissue repair by facilitating macrophage egress, inducing M2-like polarization, enhancing efferocytosis, and upregulating receptors for pro-resolving lipid mediators (SPMs) (64).

In human atherosclerosis, the functional abnormality of Tregs is not only reflected in their reduced numbers but also in distinct epigenetic and phenotypic defects. FOXP3+ Tregs within plaques exhibit elevated methylation in the CNS2 region of the FOXP3 gene, leading to the downregulation of key anti-inflammatory molecules like CTLA-4 and IL-10. Concurrently, a pro-inflammatory CXCR3+ Th1-like subset emerges, whose IFN-γ secretion is positively correlated with Ox-LDL concentration (84, 85). Furthermore, the proportion of PD-1+ Tregs in patients with acute coronary syndrome is closely associated with the risk of adverse cardiovascular events (76). Significant species differences exist between preclinical models and human disease. The regulatory effect of Tregs on macrophage polarization observed in ApoE^-^/^-^ mice is diminished in the complex human inflammatory microenvironment characterized by high lipid load and multiple complications. The potent plaque-stabilizing effect of adoptive Treg transfer in mice yields only a modest response in human clinical studies, failing to significantly reduce plaque volume. This is attributed to the longer disease course and higher degree of fibrosis in human plaques, as well as the short *in vivo* survival time of infused Tregs. In human plaques, macrophage-like VSMCs secrete higher levels of IL-6 and TNF-α, which more potently promote the conversion of Tregs into Th17 cells. Conversely, the ability of Tregs to inhibit the phenotypic reprogramming of VSMCs is relatively weak. Concurrently, novel therapeutic targets, such as CD74 blockade, which target the migrasome-APP-CD74 axis, offer new translational directions for mitigating vascular inflammation (83, 86). However, relationships between specific Tregs subsets and disease progression in humans, as well as the therapeutic efficacy of Tregs-targeted interventions, await validation in larger prospective clinical studies. The substantial species differences in plaque composition, disease chronicity, and immune cell kinetics between murine models and humans underscore the need for caution in translating these findings to clinical practice.

In summary, Tregs form a sophisticated immunosuppressive network in AS. Their dysfunction or reduction accelerates disease, making strategies to expand or restore Tregs function a promising immunotherapeutic direction.

### 3.2 Hypertension

**Figure 4:**
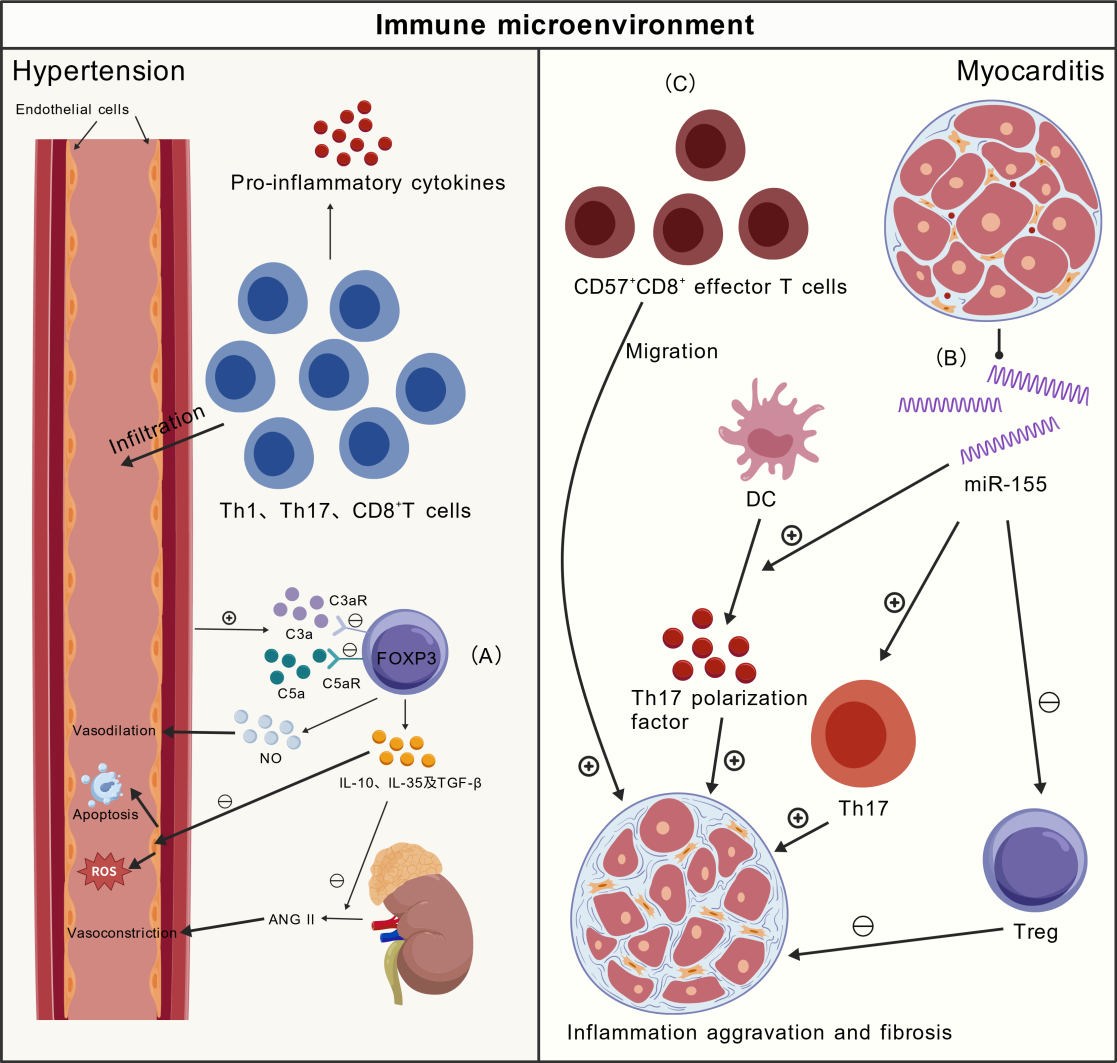
Immune mechanisms in hypertension and myocarditis. **(A)** Tregs anti-hypertensive effect: FOXP3+ Tregs secrete IL-10/IL-35/TGF-β1, reduce vascular inflammation, and promote endothelial NO production; hypertension-induced C3a/C5a impairs Tregs via C3aR/C5aR, with complement blockade as a potential strategy; **(B)** Experimental autoimmune myocarditis: Th17/Tregs imbalance and miR-155 upregulation drive disease; miR-155 inhibition alleviates myocardial injury and Th17 polarization; **(C)** Fulminant myocarditis: CXCL8^+^CD14^+^ monocytes-derived IL-10 promotes CD57^+^CD8^+^ T cell cardiac recruitment/cytotoxicity; targeting this axis mitigates immune-mediated cardiac damage. IL, interleukin; NO, nitric oxide; TGF-β1, transforming growth factor-β1.

Tregs possess potent anti-hypertensive properties. FOXP3^+^ Tregs exert protection by modulating innate and adaptive immune responses (87). They regulate hypertensive pathology by secreting IL-10, IL-35, and TGF-β1 to suppress immune overactivation. However, in hypertension, elevated complement components C3a/C5a bind to C3aR/C5aR on Tregs, directly impairing their function. This creates a vicious cycle of “hypertension-complement activation-Tregs impairment-worsened hypertension, “ amplified by upregulated C5aR on patient Tregs. This mechanism provides a novel rationale for complement blockade to preserve/restore Tregs function as an immunotargeted strategy (88). Their anti-inflammatory action reduces vascular wall inflammation, aiding normal vascular tone and pressure (89). Tregs also promote endothelial NO production for vasodilation (31). Recent studies show Fucus vesiculosus extract and its active component, Diphenyl Ketone, can reverse Th17/Tregs imbalance and restore FOXP3^+^ Tregs proportions (90) **Figure 4**.

Hypertensive immunopathogenesis involves a disrupted balance: infiltrating T lymphocytes in cardiovascular control organs induce ROS and pro-inflammatory cytokines to exacerbate hypertension (88, 91), while Tregs counteract these mechanisms. In hypertension, this protective mechanism is often impaired, perpetuating a vicious cycle. Notably, studies reveal a key paradox: Tregs therapy significantly alleviates end-organ damage in the heart and vessels but may not fully normalize blood pressure itself. This strongly suggests Tregs’ core therapeutic value lies in potent end-organ protection rather than direct blood pressure reduction (92). Future research is needed to further clarify whether Tregs exert direct antihypertensive effects, to elucidate their organ-protective mechanisms, and to explore strategies for modulating this immunoregulatory system. Such investigations may open new avenues for therapeutic development.

The protective role of Tregs in hypertension is well-established, yet their functional abnormalities exhibit significant clinical specificity in human patients. There is a clear sex-based disparity in Treg dysfunction under hypertensive conditions. Research by Pollow et al. found that menopausal women experience a significant reduction in Tregs numbers. This loss eliminates the protective effect against T cell-mediated hypertension, a phenomenon not observed in male patients. This difference is closely associated with changes in estrogen levels (89). Significant species differences between preclinical models and human disease impact the translational efficacy of Treg-based therapies. Numerous animal studies have confirmed that adoptive transfer of Tregs exerts potent vascular protective effects in hypertension models. For instance, Tregs can prevent angiotensin II-induced hypertension and vascular injury. They are also capable of inhibiting aldosterone-mediated vascular damage and oxidative stress. Conversely, the absence of Tregs exacerbates angiotensin II-induced endothelial dysfunction and vascular remodeling (93). However, the significant efficacy observed in rodent models translates only to a modest reduction in hs-CRP in human clinical studies. Achieving a substantial improvement in blood pressure control remains difficult. Although animal studies have robustly confirmed a strong correlation between Tregs/Th17 cells and hypertension, research in human hypertension remains limited (94). This substantial gap between preclinical and clinical evidence highlights the need for caution when directly extrapolating findings from animal models to humans.

### 3.3 Myocarditis

Myocarditis pathogenesis involves elevated CD4^+^ T cells and Th17/Tregs imbalance, where heightened Th17 activity overrides Tregs suppression. In experimental autoimmune myocarditis, miR-155 is upregulated in heart tissue and CD4^+^ T cells. It drives Th17/Tregs imbalance toward Th17 dominance, promoting disease. Inhibiting miR-155 reduces severity, myocardial injury, Th17 responses/polarization, and DC secretion of Th17-polarizing cytokines (95) **Figure 4**.

In fulminant myocarditis, highly cytotoxic and migratory CD57^+^CD8^+^ T cells are significantly enriched and clonally expanded. Upon cardiac migration, they exert cytotoxicity, inducing cardiomyocyte apoptosis. Elevated IL-18 drives CD57^+^CD8^+^ effector T cell differentiation. Increased pro-inflammatory CXCL8^+^CD14^+^ monocyte numbers elevate IL-18 and interact with these T cells. Disrupting this pathogenic axis—involving pro-inflammatory monocytes, IL-18 signaling, and CCR5^-^mediated cardiac recruitment—significantly alleviates fulminant myocarditis in mice (96). These findings identify CD57^+^CD8^+^ effector T cells and their upstream inflammatory signals as potential therapeutic targets for mitigating immune-mediated cardiac damage in acute and fulminant myocarditis. Further investigation is needed to determine whether targeting these pathways can yield clinical benefit.

In human fulminant myocarditis, the cytotoxicity of CD57^+^CD8^+^ T cells is significantly stronger than that in mice. The levels of perforin and granzyme B they secrete are 3.1 times higher than those in mice. The suppressive effect of Tregs on these cells is relatively weak. This species difference further impacts the translational efficacy of Treg-based therapies (96). A study simultaneously examined samples from humans and from mice infected with Coxsackievirus B3. It was found that miR-155 is consistently and strongly upregulated in both human myocarditis and susceptible mice. Its expression is primarily localized in infiltrating macrophages and T lymphocytes. Systemic inhibition of miR-155 in mice reduces the cardiac infiltration of monocytes/macrophages, decreases T lymphocyte activation, and diminishes myocardial injury during acute myocarditis. These lines of evidence indicate that miR-155 plays a key pathogenic role in both human and murine myocarditis. This provides a cross-species translational basis for therapeutic strategies targeting miR-155 (97). These observations suggest that therapeutic strategies validated in mice may require substantial optimization for human application, and that direct extrapolation of efficacy from murine to human myocarditis should be approached with caution.

In viral myocarditis, Tregs reduce acute inflammation but may also suppress antiviral immunity. Whether this suppression could lead to viral persistence, and whether such persistence might exacerbate injury, induce fibrosis, or cause long-term dysfunction. This remains to be determined experimentally.

### 3.4 Cardiac transplant rejection

Cardiac transplantation remains the gold standard for end-stage heart failure. While Tregs are a powerful tool to induce transplant tolerance, their clinical translation faces multiple challenges. Key cellular bottlenecks include: donor- vs. recipient-derived Tregs sources, and the need for antigen specificity (98); functional instability of Tregs subsets due to differential FOXP3 epigenetic regulation, limiting post-infusion efficacy (99); and potential decline in immunosuppressive function and anti-apoptotic capacity of ex vivo-expanded Tregs after infusion, impacting long-term outcomes (100). A therapeutic environment contradiction exists: conventional post-transplant immunosuppressants (e.g., calcineurin inhibitors) negatively impact Tregs numbers and function, counteracting therapeutic goals (101). Finally, manufacturing challenges persist, including limited cell sources, complex isolation/expansion protocols, and high costs inherent to adoptive cell therapy.

Current research is exploring several strategies to address these challenges: First, engineering Tregs (e.g., CAR-Tregs, antigen-specific Tregs) for enhanced specificity/potency and reduced off-target effects. Second, using engineered cytokines (e.g., IL-2 complexes, muteins) to selectively expand Tregs *in vivo* without activating effector cells. Third, optimizing immunosuppressive regimens, such as combining calcineurin inhibitors with IL-2 to rescue Tregs numbers, or leveraging Tregs expansion to enhance drugs like CTLA4Ig that preserve/promote Tregs function (102).

Regarding clinical translation, data on Tregs therapy in cardiac transplantation remain scarce. Its efficacy and generalizability in solid organ transplantation require further large-scale clinical validation.

### 3.5 Heart failure

**Figure 5:**
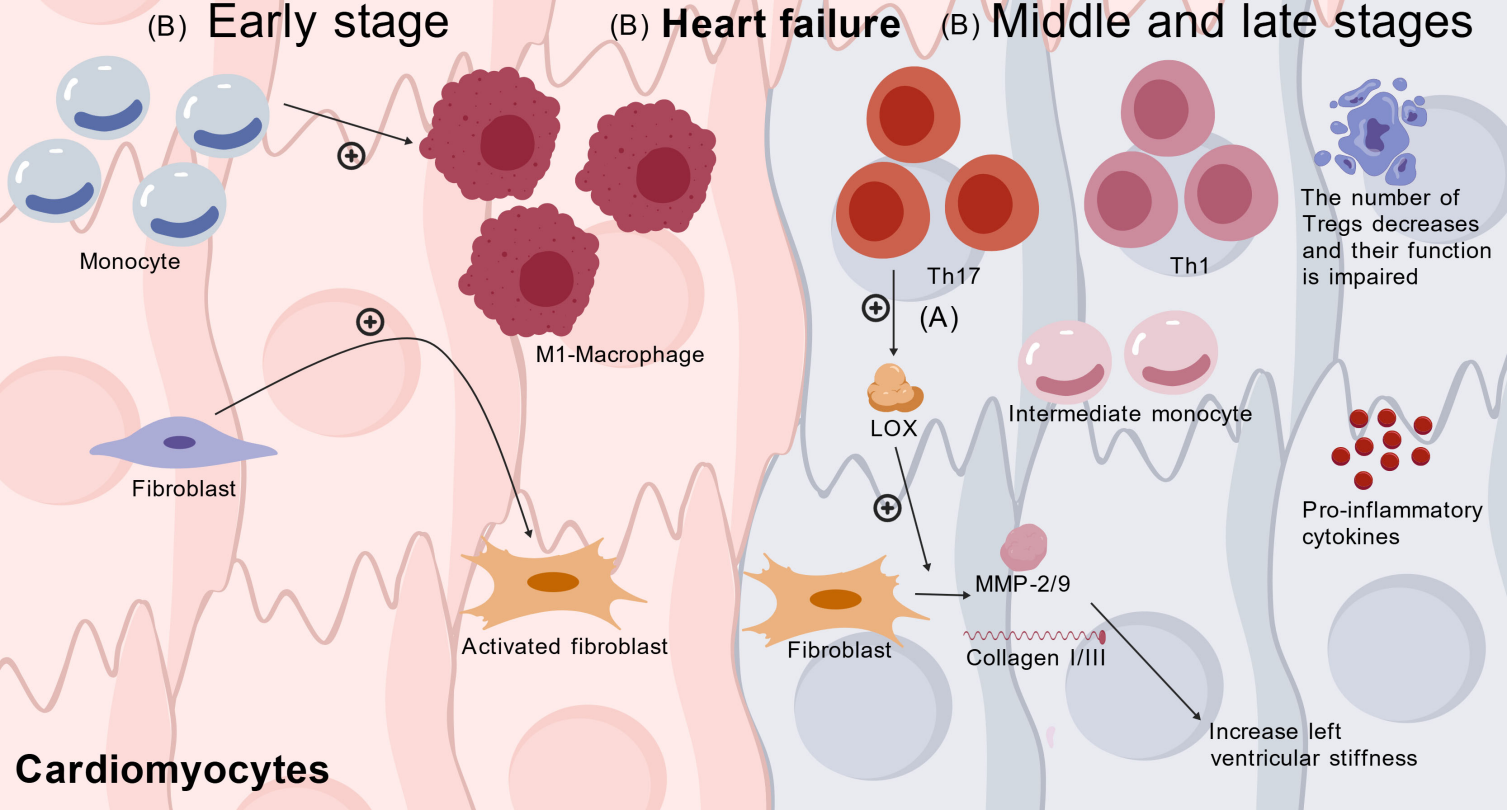
Immune-mediated inflammation and fibrosis in heart failure progression. **(A)** Th17/Tregs imbalance-driven fibrosis: Th17 upregulates LOX via IL-17/ERK1/2-AP-1 pathway; Tregs suppress LOX via IL-10/JAK1-STAT3 pathway; high LOX increases fibroblast MMP-2/9 and collagen I/III, enhancing left ventricular stiffness (53); **(B)** Temporal immune activation: Early stage: classical monocytes → M1 macrophages (acute inflammation/fibroblast activation); middle-late stages: Th1/Th17 infiltration + reduced/dysfunctional Tregs, followed by intermediate monocytes, perpetuating chronic inflammation/fibrosis and driving heart failure progression (82). CHF, chronic heart failure; LOX, lysyl oxidase; MMP, matrix metalloproteinase; IL, interleukin.

Chronic inflammation and fibrosis are key drivers of left ventricular hypertrophy, heart failure, and cardiac remodeling. Monocytes and T helper cells promote inflammation and fibrosis, whereas Tregs are thought to suppress chronic inflammation in hypertrophied myocardium (103). Th17/Tregs imbalance (increased Th17, decreased Tregs) is central to chronic heart failure (CHF). Th17 cells activate the IL-17/ERK1/2-AP-1 pathway to promote lysyl oxidase (LOX) expression, while Tregs suppress it via the IL-10/JAK1-STAT3 pathway. High LOX upregulates fibroblast expression of MMP-2/9 and collagen I/III, catalyzing collagen cross-linking and increasing left ventricular stiffness (68). In human CHF, immune cell activation follows a distinct temporal sequence: Pathological stimuli (pressure overload, hyperglycemia) initiate a local cardiac immune response. Early stages are dominated by monocyte/macrophage system activation—classical monocytes are recruited, converting to pro-inflammatory M1 macrophages to initiate acute inflammation and activate cardiac fibroblasts. As disease progresses, T cell subset imbalance becomes dominant, with increased infiltration of CD4^+^ effector T cells (Th1, Th17) alongside reduced and dysfunctional Tregs, creating a pro-inflammatory, anti-inflammatory-deficient microenvironment. Intermediate monocyte subsets then dominate, perpetuating chronic inflammation and fibrosis, collectively driving the transition from asymptomatic hypertrophy to symptomatic heart failure (103) **Figure 5**.

However, another animal study first established the central pathogenic role of CD4^+^ T cells in chronic ischemic heart failure: they drive myocardial injury and remodeling via a pathologic chain of systemic activation → local myocardial subset imbalance → memory-mediated pathogenesis. In heart failure, CD4^+^ T cells exhibit multi-organ accumulation and activation, with total CD4^+^ T cells and Th1, Th2, Th17, and Tregs subsets all significantly expanded. The Th1/Th2 ratio decreases (Th2 relative dominance), while the Th17/Tregs ratio increases (Th17 relative dominance), alongside upregulated Th2 cytokines. This local subset imbalance likely drives myocardial injury by amplifying pro-inflammatory/pro-fibrotic signals, with splenic memory CD4^+^ T cells potentially key to sustaining long-term pathology (104).

**Table 1**.

**Table 1:** The table below lists major cells and their mechanisms in heart failure  (103, 104).

Cell	Primary Pathway/effect	Mechanism
Th1	Secretes pro- inflammatory/pro- fibrotic cytokines (IFN-γ, TGF-β, IL-2)	Activates cardiac fibroblast-to-myofibroblast transformation and extracellular matrix deposition. Also modulates M1/M2 macrophage balance, potentially reinforcing a pro-inflammatory milieu. TGF-β acts here as a pro- inflammatory/fibrotic driver, suggesting its role is context-dependent.
Th17	Secretes IL-17, IL-19	Exerts similar pro-inflammatory effects.
Monocytes	Infiltration	Classical monocytes dominate acute inflammation. Intermediate and non- classical subsets favor chronic inflammation and repair/fibrosis. Intermediate monocytes contribute to sustained fibrosis by converting to pro-fibrotic M2 macrophages.
Macrophages	Infiltration	Primarily derived from peripheral monocytes, they are the critical link between microvascular inflammation and myocardial fibrosis, directly promoting extracellular matrix accumulation and myocardial stiffness.
Tregs	Secretes anti- inflammatory IL- 10	Suppresses effector T cell and macrophage activity, promoting inflammation resolution and anti-fibrosis. Their reduction or dysfunction collapses immunosuppression, exacerbating inflammation and fibrosis.
Th2	Secretes IL-4, IL- 5, IL-13, IL-18	Exerts similar anti-inflammatory effects.

In summary, substantial evidence establishes immune imbalance, characterized by an elevated Th17/Tregs ratio, as central to CHF pathogenesis. This imbalance is not isolated but embedded within a complex, temporally regulated network of interacting immune cells.

Therapeutically, cardiac resynchronization therapy (CRT) improves symptoms and remodeling but increases proportions of pro-inflammatory cytotoxic T cells producing TNF-α/IFN-γ, failing to reverse the persistent Tregs decline in CHF patients (105). In contrast, β-hydroxybutyrate treatment counteracts oxidative stress and inhibits the NLRP3 inflammasome in failing hearts. By modulating the NOX2/GSK-3β pathway to reduce GSK-3β phosphorylation at Ser9, it increases cardiac Tregs numbers, improving diastolic function, fibrosis, and remodeling (106). Oral catechin significantly suppresses immune activation, corrects IL-17/IL-10 imbalance, and reverses aberrant Th17/Tregs polarization in peripheral blood and spleen, inhibiting heart failure progression (107).

Immunopathological studies of human heart failure provide key evidence supporting the core concepts derived from preclinical models. The Th17/Treg imbalance has been directly validated in patients with chronic heart failure (CHF). A study of 100 CHF patients showed that, compared to healthy controls, these patients exhibit a significant Th17/Treg imbalance (characterized by an increased Th17/Treg ratio) and a Th1/Th2 imbalance (skewed toward Th1). Furthermore, these immune imbalance markers are significantly correlated with left ventricular end-diastolic dimension (LVEDd) and brain natriuretic peptide (BNP) levels. They are also negatively correlated with left ventricular ejection fraction (LVEF) (108). Human studies have also expanded our understanding of immune pathology in new dimensions. Youn et al. discovered a significantly increased proportion of CD4^+^CD57^+^ senescent T cells in patients with acute heart failure. These cells exhibit a more pronounced inflammatory profile, characterized by the production of IFN-γ and TNF-α. Both their baseline levels and the extent of their increase over a 6-month follow-up period were significantly correlated with the occurrence of adverse cardiovascular events in patients (109). Additionally, research by Zhang et al., focusing on dilated cardiomyopathy (DCM), utilized multi-algorithm immune infiltration analysis and validated by flow cytometry. They found a significantly increased proportion of CD4^+^ effector memory T cells (CD4^+^ TEM) in DCM patients. This finding suggests that, beyond the Th17/Treg imbalance, T cell terminal differentiation and memory responses also represent potential therapeutic targets (110). These findings show that we need more human−based studies to reflect the real diseases and complications that cannot be seen in animal experiments.

However, these findings raise a deeper question: while relative/absolute Tregs deficiency is considered foundational to CHF immunodysregulation, cases exist where Tregs numbers increase but function remains inadequate, failing protection or even promoting inflammation (111). This suggests that assessing immune status by cell count alone may be insufficient, and that evaluating Tregs function and quality, in addition to quantity, is likely important for understanding their role in heart failure. A key consideration in current research is Tregs plasticity—while they maintain immune homeostasis under physiological conditions, they can adopt pro-inflammatory phenotypes in specific inflammatory microenvironments. This plasticity represents a significant hurdle for the development of Tregs-targeted therapies. Consequently, subsequent investigations should aim to identify the signals that drive Tregs functional conversion and to explore strategies for maintaining their protective phenotype. The next section will delve into Tregs functional complexity and its precise upstream regulation in heart failure.

## 4 Beyond immunosuppression: the complexity and novel regulatory dimensions of Tregs function

While the protective role of Tregs in CVDs is well-established, observations that immunosuppressive function can fail despite normal or even elevated Tregs numbers suggest that the traditional model of numerical deficiency alone is insufficient to explain disease pathogenesis. Accumulating evidence indicates that the immunopathology of CVDs involves not only Tregs deficiency but also functional alterations among Tregs subsets, tissue-specific adaptation, and metabolic interactions with the inflammatory microenvironment (104). This reveals remarkable functional heterogeneity and plasticity within the Tregs population (112, 113). During atherosclerosis progression, Tregs have been observed to convert into pro-inflammatory T-cell subsets (114). These findings shift the focus from assessing Tregs quantity alone toward understanding which Tregs subsets, in which microenvironmental contexts, perform what functions. This section reviews the functional diversity of Tregs, their regulation by metabolic checkpoints, and the neuro-immune-cardiovascular axis as an upstream regulatory pathway. It also discusses how these insights may inform the development of next-generation therapeutic strategies, including antigen-specific Tregs adoptive transfer, metabolism-targeting approaches, and bioelectronic interventions, and considers the potential of Tregs-targeted precision immunotherapy for CVDs management.

### 4.1 Tissue-resident Tregs

This evolving understanding has refocused research attention from circulating Tregs toward the complex tissue microenvironment. Tissue-resident Tregs (trTregs) exemplify Tregs diversity and environmental dependency. They not only contribute to local immune homeostasis but also acquire specialized functions at specific anatomical sites that extend beyond immunosuppression to include tissue homeostasis and repair. Their core characteristics, functional duality, plasticity, and role in CVDs are elaborated below.

As a specialized Treg subset, trTregs exhibit unique tissue-specific phenotypic features enabling precise inflammatory control and tissue homeostasis maintenance at their residency site. Beyond their well-established immunosuppressive role, accumulating evidence highlights their central function in regulating tissue injury response and driving regeneration (115). Beyond classic markers (CD4, CD25, Foxp3), trTregs display tissue-specific transcriptional profiles and functional molecules—such as antigen-specific TCRs and the IL-33 receptor ST2 encoded by Il1rl1—conferring specialized local functions (116). Their tissue homing and residency are regulated by multiple factors: transcription factors (e.g., Hobit, Blimp-1, KLP2) (117, 118); chemokine receptors (e.g., CCR4, CCR6, GPR15) and their ligands (119–121); adhesion molecules (e.g., LFA-1, α4β7 integrin, LAYN) (122–124); and tissue microenvironment cytokines (e.g., IL-33, IL-2) and metabolites (125, 126).

The heart harbors reparative trTregs that reside in the parenchyma to maintain local homeostasis. Post-myocardial infarction, the heart recruits tissue-specific Tregs with a unique transcriptome and pro-repair phenotype, showing upregulated CTLA-4 and Klrg-1 expression. They primarily originate from circulation, with local expansion and conversion of conventional T cells as secondary sources; the IL-33/ST2 axis regulates their cardiac enrichment by promoting Tregs expansion. These Tregs highly express molecules like Sparc and Areg, directly repairing the heart post-MI by increasing scar collagen content and promoting collagen maturation to maintain integrity, extending beyond mere immunosuppression (127). These findings identify cardiac-resident Tregs as participants in endogenous repair processes following injury, providing a cellular rationale for investigating Tregs subset-targeted approaches in heart failure.

However, this functional specialization also confers functional duality in tissue repair and fibrosis. While trTregs can facilitate repair by suppressing excessive inflammation, they may also contribute to disease progression in specific pathological contexts.

In chronic ischemic heart failure, CD4^+^Foxp3^+^ Tregs expand in phases post-MI, persisting beyond early wound healing. These Tregs exhibit pro-inflammatory Th1-like features, expressing IFN-γ, TNF-α, and TNFR1. Via TNFR1-dependent pathways, direct endothelial contact, and CCL5/CCR5 signaling, they promote immune activation, inhibit angiogenesis, and drive pathological left ventricular remodeling. Selective ablation or depletion of these aberrant Tregs reverses remodeling and dysfunction, reduces inflammation and fibrosis, enhances angiogenesis, and the repopulated Tregs regain normal function (111). This study clarifies a pathogenic Tregs role in chronic ischemic heart failure, highlighting the need to scrutinize Tregs functional diversity and conversion.

Further illustrating trTregs functional duality in chronic injury and fibrosis: they exert anti-fibrotic effects by suppressing inflammation and fibroblast activation. CCl4-induced chronic hepatitis selectively expands hepatic Tregs, inhibiting fibrosis, while Tregs depletion in CCl4 models exacerbates liver pathology (128). Bleomycin-induced lung injury generates an IL-33-dependent Tff1^+^ Tregs subset that attenuates pulmonary fibrosis by modulating macrophage phenotypic switching, suppressing their pro-inflammatory phenotype. Although Tff1 itself is not the critical anti-fibrotic factor, loss of the Tff1+ Tregs subset significantly worsens pulmonary fibrosis (129). Conversely, in specific pathological settings, Tregs can secrete AREG, TGF-β, and PDGF to promote fibroblast proliferation and epithelial-mesenchymal transition, thereby exacerbating fibrosis (130, 131). This seemingly paradoxical output underscores that trTregs function is not fixed but critically depends on subset identity, the local tissue microenvironment, and disease stage (132, 133).

TrTregs functional duality prompts inquiry into its origins within a given tissue—whether linked to inflammatory cues, metabolic factors, and local metabolite signaling—the underlying mechanisms, and the potential to target these pathways to reverse disease. The deeper biological basis lies in a fundamental Tregs property: functional plasticity. This means Tregs can undergo dynamic, even fundamental, reprogramming under strong inflammatory or metabolic stress signals. While this plasticity provides immune system flexibility, in chronic diseases like atherosclerosis it poses a serious risk, converting protective Tregs subsets into pathogenic effector cells—a major challenge for Tregs-targeted therapies.

### 4.2 Tregs plasticity and functional conversion

Conventional CD4^+^CD25^+^FOXP3^+^ Tregs offer broad therapeutic benefits in CVDs. However, Tregs plasticity was proposed earlier (134), but related research remains sparse, and this plasticity can be detrimental in CVDs. Within the inflammatory CVDs microenvironment, protective Tregs can convert into pathogenic cells, exacerbating disease progression—a major immunotherapy challenge.

Physiological adaptation and pathological conversion represent the core dichotomy of Tregs functional plasticity. This dichotomy directly determines their protective or pathogenic roles in CVDs. The boundary between these two states is collectively defined by microenvironmental signals, phenotypic stability, and functional output (7). Although conceptually useful, the dichotomy between physiological adaptation and pathological conversion should be viewed as a working model rather than a rigid classification. It reflects current understanding of Treg plasticity under distinct microenvironmental cues, but the boundaries between these states are often context-dependent and may overlap in complex disease settings.

Physiological adaptation is a protective remodeling of Tregs in response to the demands of a physiological microenvironment. Its core features are stable FOXP3 expression and retained anti-inflammatory function. These adaptive changes serve to maintain local homeostasis. The driving signals are often physiological factors, such as IL-33 and gut microbiota-derived short-chain fatty acids (SCFAs). These enhance the tissue-specific functions of Tregs by activating pathways like AMPK and STAT5. For example, cardiac-resident Tregs highly express ST2 and Areg, facilitating tissue repair after myocardial infarction by promoting collagen maturation (127). In vascular tissue, Tregs utilize the CD39/CD73 pathway to convert ATP into adenosine. This inhibits low-grade endothelial inflammation and maintains vascular homeostasis (54). At the metabolic level, Tregs under physiological conditions rely on oxidative phosphorylation and fatty acid oxidation for energy. FOXP3 confers a survival advantage in low-glucose microenvironments by inhibiting glycolysis. This ensures the persistence of their immunosuppressive function (135).

Pathological conversion is a pathogenic phenotypic transformation of Tregs driven by pathological signals such as chronic inflammation and metabolic disorders. The hallmark is the loss or functional inhibition of FOXP3 expression, coupled with the acquisition of Th1/Th17-like pro-inflammatory characteristics. Driving factors include oxidized low-density lipoprotein (Ox-LDL), pro-inflammatory cytokines (IL-6, TNF-α), and complement components (C3a/C5a). These factors disrupt Treg stability by activating pathways such as PI3K/Akt/mTOR and NF-κB (85). For instance, in atherosclerosis, ApoB100-reactive Tregs lose FOXP3 expression under hypercholesterolemia. They convert into effector cells secreting IFN-γ and IL-17, thereby exacerbating plaque inflammation (67). In chronic ischemic heart failure, myocardial Tregs display a Th1-like phenotype and highly express TNFR1. This drives myocardial fibrosis by inhibiting angiogenesis (111). At the epigenetic level, pathological signals induce the expression of the DNA methyltransferase Dnmt3a. This leads to hypermethylation of the CNS2 region in the FOXP3 gene, further stabilizing their pathogenic phenotype (136).

The functional plasticity of Tregs is context-dependent. Their fate is determined by the tissue microenvironment, disease stage, and local signals. At the tissue-specific level, in myocardial infarction, cardiac Tregs promote tissue repair (137). While in atherosclerosis, Tregs can lose Foxp3 and acquire a Th1-like phenotype (138). At the disease stage level, the role of Tregs can change over time. In the early phase of myocardial infarction, Tregs are protective. However, in chronic ischemic heart failure, some Treg subsets become dysfunctional and express pro-inflammatory markers, which can worsen cardiac remodeling (137). At the signal combination level, the same cytokine can have opposite effects. TGF-β induces Tregs differentiation without inflammation but promotes Th17 differentiation in the presence of IL-6 (66). These observations indicate that Tregs function is dynamically regulated by environmental cues.

The distinction between these states has potential therapeutic implications. Physiological adaptation is associated with maintaining immune tolerance and promoting tissue repair, and may be reversible upon removal of physiological signals. Pathological conversion, by contrast, is associated with amplifying inflammation and exacerbating tissue damage, and may be less readily reversible. This framework suggests potential therapeutic approaches: for insufficient physiological adaptation, strategies such as supplementing SCFAs or low-dose IL-2 are under investigation to support Tregs function; for pathological conversion, approaches combining FOXP3 stabilizers with inhibitors of pro-inflammatory signals are being explored to mitigate pathogenic drift.

Despite growing evidence of Tregs plasticity in cardiovascular diseases, several key questions remain unresolved. The molecular switches that determine whether Tregs undergo protective adaptation or pathogenic conversion are unclear. How inflammatory signals regulate epigenetic modifications at the Foxp3 locus is not fully understood (139). Whether converted Tregs can be reversed to a stable phenotype remains unknown. We believe that future studies integrating lineage tracing and single-cell epigenomics may help address these questions.

Pathogenic autoimmunity in atherosclerosis originates from an initially protective autoimmune response. Apolipoprotein B100 (apoB) drives pathogenic Th1 cell generation and exacerbates atherosclerotic lesions. Healthy mice harbor a natural ApoB-reactive CD4^+^ T cell (ApoB^+^) population exhibiting a Tregs-like transcriptome yet a mixed lineage phenotype (Tregs/Th17/Th1). Driven by hypercholesterolemia in Apoe^-^/^-^ mice, this population clonally expands and undergoes marked phenotypic conversion, losing Tregs features (FoxP3 loss, forming exTregs) while converting into Th1/Th17-like pathogenic effector T cells secreting IFN-γ and IL-17, failing to counteract AS. Coronary Artery Disease (CAD) patients’ circulation contains ApoB-reactive T cells with a Th1/Th17 phenotype, demonstrating conversion from a protective to a pathogenic immune response. These findings implicate functional instability of ApoB-reactive Tregs in disease pathogenesis and suggest them as potential therapeutic targets (67).

Another study has characterized Tregs phenotypic conversion in atherosclerosis Atherosclerosis promotes Tregs plasticity, converting a CXCR3^-^expressing phenotype into a dysfunctional intermediate subtype co-expressing Tregs markers and Th1 features (e.g., IFN-γ secretion, CCR5 expression). These Th1-like Tregs originate from bona fide Tregs, not effector T cells, functionally losing immunosuppressive capacity and failing to ameliorate AS lesions. Their unique transcriptome shows downregulated Tregs stability/function genes, involving dysregulation of IFN-γ, IL-2, and TCR signaling pathways, ultimately converting them from protective cells to promoters of arterial inflammation and progression (85).

In CVDs pathogenesis, Tregs plasticity and functional conversion are closely regulated by the disease microenvironment and aberrant inflammatory signals. An experiment demonstrates Tregs functional plasticity in specific inflammatory settings: immunosuppressive CD4^+^CD45RA^-^CD25highCCR6^+^HLA^-^DR^-^FOXP3^+^ Tregs can be induced by IL-1β and IL-6 to produce the pro-inflammatory cytokine IL-17, a process inhibited by TGF-β. Single-cell cloning confirmed that the same Treg cell holds dual potential for IL-17 secretion and immunosuppression; functional output depends on microenvironmental signals: IL-17 production coincides with reversible loss of suppressive function while maintaining FoxP3 expression (140). *In vivo*, Tregs in inflammatory bowel disease and rheumatoid arthritis exhibit plasticity toward IL-17 secretion and Th17 conversion (141, 142). These observations suggest an inflammation-driven mechanism whereby local IL-17 production by Tregs may transiently weaken their suppressive function, potentially contributing to inflammation in specific contexts.

Regarding the plasticity of Tregs, the dual function of TGF-β is a core manifestation of their functional heterogeneity. Although TGF-β is widely recognized as a core cytokine mediating immunosuppression by Tregs, numerous studies have confirmed that its function exhibits significant duality. TGF-β plays a balancing role in immune regulation: it is crucial for maintaining immune tolerance, yet it can also drive inflammatory responses under specific conditions. Specifically, TGF-β plays a critical role in determining the differentiation fate of naive CD4^+^ T cells. In the absence of inflammatory signals, TGF-β induces FOXP3 expression and promotes the differentiation of anti-inflammatory Tregs. However, in the presence of pro-inflammatory cytokines such as IL-6 or IL-21, TGF-β synergizes with these signals to activate STAT3, thereby driving the differentiation of pro-inflammatory Th17 cells (143, 144). This functional duality of TGF-β is precisely a core reflection of the functional heterogeneity of Tregs.

This functional dichotomy is also reflected in the distinct subsets of Tregs and their stability. The generation of peripherally induced Tregs is highly dependent on TGF-β signaling, but their lineage stability is poor. Within a persistent inflammatory microenvironment, such as in atherosclerotic plaques, these Tregs are highly susceptible to phenotypic conversion. They lose FOXP3 expression and acquire the characteristics of pro-inflammatory effector cells like Th17 cells. In this context, TGF-β originally secreted by Tregs, under the influence of the local inflammatory milieu, may instead promote the differentiation of other T cells into Th17 cells via paracrine signaling. This, in turn, exacerbates vascular inflammation and disease progression (145). Therefore, the dual function of TGF-β profoundly reveals that Tregs are not a functionally homogeneous population. Their ultimate immunological effect depends on complex signal integration and the environmental context.

In summary, Tregs plasticity has been observed in various CVDs immunopathologies and experimental models. These observations challenge the traditional view of Tregs stability and suggest that immune dysregulation in CVDs involves not only insufficient Tregs numbers but also alterations in Tregs quality and function. Key questions for further investigation include: distinguishing physiological adaptation from pathological conversion; identifying additional Tregs phenotypic transitions and underlying molecular mechanisms in cardiovascular diseases; developing strategies to maintain Tregs phenotype stability in diseased microenvironments; and exploring approaches to modulate local Tregs plasticity for therapeutic benefit. A current limitation is that much of the evidence is descriptive or correlative; the molecular mechanisms driving Tregs fate conversion (e.g., epigenetic reprogramming, metabolic checkpoints) remain to be identified. Future studies employing lineage tracing, CRISPR screening, and other advanced techniques may help elucidate these processes.

### 4.3 Metabolic checkpoints

T cell activation and differentiation are tightly coupled to profound metabolic reprogramming. Cardiovascular lesions (e.g., atherosclerotic plaques) often exhibit a metabolically stressed state of hypoxia, high lactate, and high lipids. Nutrient availability and specific metabolic pathways constitute metabolic checkpoints determining Tregs and effector T cell survival and function (146, 147).

#### 4.3.1 Nutrient competition and immunometabolic regulation

During immune responses, different T cell types utilize nutrients distinctly; this metabolic competition forms a novel “metabolic checkpoint” central to immune balance regulation. Effector T cells (Teff) rapidly upregulate glucose uptake and glycolysis upon activation (148–150). Conversely, Tregs exhibit lower glucose uptake capacity, relying primarily on oxidative phosphorylation and fatty acid oxidation for energy (151). In glucose-restricted environments, Teff cells are more susceptible to metabolic stress, whereas Tregs have a survival advantage (152). In CVDs like atherosclerosis and ischemic heart failure, lesions often present a metabolically stressed microenvironment of hypoxia, high lactate, and lipid enrichment, generating metabolites like lactate and pro-inflammatory factors (153). In this microenvironment, heavy glucose consumption by Teff cells can limit their own function while promoting Tregs stability/expansion (150). The Tregs transcription factor Foxp3 regulates T cell metabolism by inhibiting Myc signaling and glycolysis while enhancing oxidative phosphorylation and NAD regeneration, granting Tregs a metabolic advantage in low-glucose, high-lactate settings and tolerance to L-lactate inhibition. For Teff cells, L-lactate influx and reverse lactate dehydrogenase reaction block NAD regeneration and inhibit glycolysis, making Tregs function dominant in such conditions (135).

The competition for nutrients like glucose and lactate and the resulting metabolic checkpoint between Tregs and Teff cells are governed by intricate molecular signaling networks. Understanding these interactions is key to elucidating Tregs metabolic regulation and provides a direct rationale for developing metabolism-targeted immunotherapies.

#### 4.3.2 PI3K/Akt/mTOR and AMPK pathways as targets for metabolic intervention

Key metabolic signaling pathways are central to Tregs subset metabolic preferences. The PI3K/Akt/mTOR pathway is a major signaling axis promoting Teff glycolysis and inhibiting Tregs differentiation (154, 155).

T cell activation, initiated by TCR-antigen binding and CD28 co-stimulation, recruits and activates PI3K. Activated PI3K catalyzes PIP2 to PIP3 at the plasma membrane (156). PIP3 acts as a second messenger, recruiting PDK1 and Akt to the membrane (157). Akt requires dual phosphorylation at Thr308 (by PDK1) and Ser473 (by mTORC2) for full activation (158), subsequently phosphorylating TSC2 to inhibit the TSC complex, allowing Rheb to remain active and activate mTORC1 (159). Amino acids also facilitate mTORC1 activation via lysosomal Rag GTPases (160). Activated mTORC1 promotes protein synthesis via phosphorylating S6K1/4E-BP1 (161). It upregulates lipid synthesis via SREBP1/PPARγ, phosphorylates ULK1 to inhibit autophagy (162), and upregulates HIF-1α to promote glycolysis (Warburg effect). The mTOR pathway precisely regulates CD4^+^ T cell differentiation: mTORC1 is essential for Th1/Th17 differentiation; mTORC2 favors Th2; inhibiting mTORC1 promotes fatty acid oxidation and Tregs generation (163). Notably, its overactivation disrupts the metabolic milieu for Tregs generation/function, breaking immune balance and inducing autoimmunity or excessive inflammation (164).

The AMPK pathway opposes PI3K/Akt/mTOR function. As a cellular energy sensor, AMPK activates during energy stress (e.g., glucose scarcity, high AMP/ATP ratio), primarily initiating catabolism to restore energy homeostasis (165). Activated AMPK phosphorylates and inhibits ACC1, reducing fatty acid synthesis while promoting oxidation and Tregs differentiation. It also phosphorylates/activates ULK1 to initiate autophagy, directly antagonizing mTORC1-mediated autophagy inhibition (166, 167). In T cell differentiation, AMPK activation promotes Tregs generation/function stability via enhancing fatty acid oxidation and potentially inhibiting mTORC1, contrasting with mTOR-driven Teff differentiation (167, 168). Furthermore, AMPK signaling enhances mitochondrial oxidative metabolism in CD8^+^ T cells, aiding memory T cell development and longevity (169).

The PI3K/Akt/mTOR and AMPK pathways antagonistically integrate growth signals and energy status to determine T cell fate: under nutrient sufficiency, the former drives Teff clonal expansion/function; during energy restriction, the latter activates, inhibiting mTORC1 and initiating catabolism to promote memory T cell and Tregs differentiation. Consequently, targeting metabolic checkpoints is a novel therapeutic strategy: inhibiting glycolysis (e.g., with 2-deoxyglucose) suppresses Teff function, alleviating experimental autoimmune encephalomyelitis (152); the mTOR inhibitor rapamycin promotes Tregs expansion and inhibits Teff responses, effective against transplant rejection, autoimmunity, and atherosclerosis in animals (170); β-hydroxybutyrate increases cardiac Tregs via modulating NOX2/GSK-3β pathway, improving diastolic function, fibrosis, and remodeling in heart failure patients (106); small molecules like Bz-423 selectively target mitochondrial metabolism, inducing apoptosis of overactive alloreactive T cells (171); targeting the AMPK pathway can also modulate immune responses via metabolic balance.

In summary, nutrient competition between Tregs and Teff cells represents a potential metabolic checkpoint; intervening in these pathways may offer approaches for modulating immune balance in autoimmune and chronic inflammatory diseases.

#### 4.3.3 Lipid metabolic reprogramming

Upon activation, T cells reprogram cholesterol metabolism: inducing the SREBP pathway to promote *de novo* cholesterol synthesis for proliferation, while downregulating the liver X receptor (LXR) pathway and its targets (e.g., cholesterol transporter ABCG1) to limit efflux and maintain intracellular cholesterol levels. LXR is a key metabolic checkpoint; activation inhibits T cell proliferation by promoting cholesterol efflux (172). Conversely, inhibiting cholesterol esterification enzyme ACAT1 increases plasma membrane free cholesterol, enhancing TCR clustering/signaling and boosting CD8^+^ T cell effector function (173). In pathologies like atherosclerosis, Ox-LDL disrupts immune balance: activating the Fas/FasL/Caspase-3 pathway to induce Tregs apoptosis, while initiating the NF-κB pathway to promote Th17 proliferation, causing Th17/Tregs imbalance and increasing plaque instability (153). Thus, ACAT1 is both an established atherosclerosis target and a potential cancer immunotherapy target (173). These findings establish the central role of lipid metabolism in regulating T cell and Tregs function, providing key targets for metabolic intervention in immune-related diseases.

#### 4.3.4 Tryptophan metabolism and immune tolerance establishment

In immunometabolism, the availability of specific amino acids and their metabolites crucially shape adaptive immune responses. The tryptophan (Trp) metabolic pathway exemplifies this link between the local microenvironment and systemic immune tolerance. It operates via two main routes: the indoleamine 2, 3-dioxygenase 1 (IDO1)-mediated Trp/kynurenine (Kyn) degradation pathway and the microbe-mediated indole derivative pathway. Trp precisely regulates T cell differentiation, function, and fate, maintaining a delicate balance between immune activation and tolerance.

##### 4.3.4.1 The IDO1-mediated Trp/Kyn pathway

In chronic inflammatory sites like atherosclerotic plaques, tolerogenic dendritic cells and macrophages highly express IDO1, catalyzing Trp degradation to Kyn. This circuit exerts dual immunosuppressive effects. First, IDO1 depletes local Trp, causing uncharged tRNA accumulation that specifically activates GCN2 kinase in T cells, triggering the integrated stress response, leading to Teff proliferation arrest and anergy induction (174). Second, under low Trp/high Kyn conditions, a GCN2-dependent mechanism downregulates the T cell receptor CD3ζ chain, inhibiting TCR signaling and suppressing CD8^+^ T cell function. Meanwhile, CD4^+^ T cells upregulate Foxp3 via GCN2 and autocrine TGF-β-dependent mechanisms, converting into suppressive Tregs (175).

##### 4.3.4.2 Microbial indole metabolism

Gut microbiota convert dietary Trp into ligands for the aryl hydrocarbon receptor (AhR). AhR regulation of T cell differentiation is ligand-dependent: binding to environmental TCDD, endogenous ITE, or microbiota-derived indoles promotes Tregs differentiation and immune tolerance; binding to photoproduct FICZ favors pro-inflammatory Th17 differentiation, exacerbating inflammation (176, 177).

Therefore, targeting the Trp pathway to enhance tolerance is a promising strategy. Dietary or microbial interventions to moderately elevate levels of AhR-activating microbiota metabolites (e.g., indole-3-propionic acid) can activate AhR, boost Tregs function, foster a tolerogenic immune microenvironment, and achieve safe, sustained anti-inflammatory effects.

These metabolic checkpoint mechanisms reveal how the local AS plaque microenvironment directly shapes and constrains Tregs fate and function “downstream” via nutrient availability and metabolic pathways. However, immune regulation is a highly integrated, multi-level system. Beyond local metabolic signals, a CNS-led, “top-down” systemic regulatory network exists: the neuro-immune-cardiovascular axis. Via specific neural circuits and neurotransmitters, this axis can remotely control Tregs function systemically (including in key immune organs like the spleen) from the “upstream, “ offering a novel systemic perspective on cardiovascular immune homeostasis and opening innovative therapeutic avenues like bioelectronic medicine.

### 4.4 The neuro-immune-cardiovascular axis: a novel “upstream” pathway regulating Tregs

As a core system for systemic Tregs regulation, the neuro-immune-cardiovascular axis operates via two key pathways, providing a “top-down” regulatory perspective on cardiovascular immune homeostasis.

First is the cholinergic anti-inflammatory pathway: cholinergic neurons from the brainstem dorsal motor nucleus project via the vagus nerve to the celiac-superior mesenteric ganglion, enhancing splenic nerve activity and inhibiting TNF production. Vagus nerve fibers form varicosities around splenic nerve cell bodies, activating the splenic nerve via cholinergic transmission (178). The splenic nerve is adrenergic, releasing norepinephrine that stimulates splenic memory phenotype CD4^+^ T cells (CD44high, CD62Llow) to express choline acetyltransferase (ChAT), synthesizing and releasing acetylcholine. This acetylcholine acts on macrophage α7 nicotinic acetylcholine receptors (α7nAChR), inhibiting pro-inflammatory cytokine production like TNF-α. Pathway integrity relies on T cells: vagus stimulation fails to suppress endotoxemia-induced TNF-α in T-cell-deficient nude mice, a function restored by adoptive transfer of ChAT^+^ T cells. T cell activation upregulates ChAT and enhances acetylcholine release. A direct link to Tregs remains unclear, though some activated ChAT^+^CD4^+^ T cells secrete IL-10, suggesting potential regulatory function (179).

The second pathway involves the sympathetic nervous system, which releases catecholamines like norepinephrine acting on β2-adrenergic receptors (β2AR) expressed on Tregs. β2AR activation increases intracellular cAMP in Tregs, triggering PKA-dependent CREB phosphorylation, thereby enhancing Tregs suppressive function: inhibiting IL-2 production by reactive CD4^+^ T cells, promoting naive T cell conversion to induced Tregs, and upregulating Tregs surface CTLA-4 in a PKA-dependent manner to strengthen immunosuppressive activity (180). Conversely, chronic βAR signaling inhibits CD8^+^ T cell proliferation (APC-independent), IFN-γ production, and cytotoxic killing, and diminishes efficacy of CD8^+^ T cell-targeted immunotherapies (e.g., anti-PD-1, anti-4-1BB antibodies) (181). Thus, adrenergic signaling exerts dual immune effects: enhancing Tregs suppression while inhibiting CD8^+^ T cell effector function.

The identification of this neuro-immune-cardiovascular axis suggests that approaches to immune modulation may expand to include bioelectronic strategies such as neural stimulation. Clinical translation of these approaches faces several challenges, including how to specifically modulate splenic Tregs without systemic sympathetic or parasympathetic activation, and how individual variability in neural function, underlying diseases, or circadian rhythms might influence therapeutic effects. Addressing these questions will require interdisciplinary collaboration among immunology, neuroscience, and bioengineering.

In summary, the functional plasticity of tissue-resident Tregs, their regulation by metabolic checkpoints, and neuro-immune axis control reveal their biological complexity. Future therapies may need to move beyond simply increasing Tregs numbers, focusing instead on precise regulation of their functional stability, tissue targeting, and metabolic crosstalk with the microenvironment.

### 4.5 CD8^+^ regulatory T cells: a new dimension in cardiovascular immune regulation

Traditionally, research on regulatory T cells has focused primarily on CD4^+^FOXP3^+^ Tregs. However, a growing body of evidence indicates that immunosuppressive functions are also present within T cell subsets expressing the CD8 molecule. Phenotypically, CD8^+^ Tregs exhibit high heterogeneity and can express markers such as CD25, FOXP3, and CD122. Some of these subsets exert their suppressive functions independently of FOXP3, utilizing IL-10 or TGF-β instead. Similar to CD4^+^ Tregs, CD8^+^ Tregs can also inhibit the proliferation and function of effector T cells. They play a significant role in maintaining immune homeostasis and self-tolerance (182, 183).

In the context of atherosclerosis, CD8^+^ T cells have long been considered to play a primarily pathogenic role. They contribute to the formation of the necrotic core and plaque instability through their cytotoxic effects. However, CD8^+^ T cells do not exclusively exert pro-atherogenic effects in atherosclerosis. Specific subsets of these cells can mediate protective, anti-atherogenic effects. This underscores the bidirectional and complex nature of their role in the pathological process of atherosclerosis. Characterized phenotypically by the expression of CD25 and other surface markers, these CD8^+^ T cells possess clear immunosuppressive functions. They can negatively regulate the activation and proliferation of effector T cells. Furthermore, they effectively mitigate the inflammatory response within atherosclerotic plaques. The expression of CD8^+^CD25^+^ T cells was detected in apoE^-^/^-^ atherosclerotic model mice. This cell population exhibits typical suppressive phenotypic and functional characteristics and can significantly inhibit the proliferation of splenic CD4^+^ T cells. Adoptive transfer experiments further confirmed that this cell population could significantly alleviate the severity of atherosclerotic lesions in recipient mice. These CD8^+^ T cells regulate the immune response by secreting anti-inflammatory cytokines. They promote the resolution of inflammation and maintain homeostasis, ultimately contributing to the stabilization of atherosclerotic inflammatory lesions (182, 184). Research by Clement et al. demonstrated that Qa-1-restricted CD8+ Tregs exert a clear anti-atherosclerotic protective effect in an apoE^-^/^-^ mouse model. They achieve this by targeting and regulating the follicular helper T cell-germinal center B cell axis. This action inhibits the formation of aortic tertiary lymphoid organs and vascular inflammation. The functional status of this cell subset is directly correlated with the severity of atherosclerotic lesions (185). Another study identified a protective CD39^+^ CD8^+^ Treg subset in both the apoE^-^/^-^ mouse model of atherosclerosis and human atherosclerotic plaque specimens. This subset exerts anti-inflammatory effects by inhibiting the secretion of pro-inflammatory cytokines such as IFN-γ and TNF-α. Consequently, it can limit excessive intra-plaque inflammation and maintain local immune homeostasis within the plaque (186).

In the field of heart transplantation, the role of CD8^+^ Tregs is more clearly defined. In a model of transplantation tolerance induced by blocking the CD40-CD40L signaling pathway, CD8+CD45RClow Tregs are the core cell population mediating long-term graft survival. Their suppressive function depends on the secretion of FGL2 and its binding to the FcγRIIB receptor. This mechanism provides a theoretical basis for the clinical application of CD8^+^ Tregs (187).

Currently, therapeutic strategies targeting CD8^+^ T cells are at a critical stage of translation from basic research to clinical application. Preclinical development of the first therapeutic anti-CD8 monoclonal antibody (PLG101) is underway. It aims to treat diseases characterized by excessive CD8^+^ T cell activation by depleting or modulating the activity of pathogenic CD8^+^ T cells. These diseases include cardiovascular conditions such as ischemic heart failure and fulminant myocarditis (188). However, given the coexistence of pathogenic cytotoxic subsets and protective regulatory subsets within CD8^+^ T cells, achieving precise regulation is challenging. The key focus of future research will be to suppress effector CD8^+^ T cells while preserving or expanding CD8^+^ Tregs. In-depth analysis of the differentiation mechanisms, antigen specificity, and interaction networks of CD8^+^ Tregs within the cardiovascular disease microenvironment will lay the foundation for developing more selective immunotherapies.

## 5 Treatment

Tregs immunotherapy represents an approach that differs from conventional drugs or surgery by targeting chronic immune inflammation with the goal of restoring immune tolerance and promoting an anti-inflammatory microenvironment. Its advantages include functioning as a “living drug” with long-term survival and dynamic anti-inflammatory activity that actively promotes tissue repair. Antigen-specific or tissue-targeting strategies offer greater precision and safety, potentially enabling disease reversal and long-term homeostasis.

**Figure 6:**
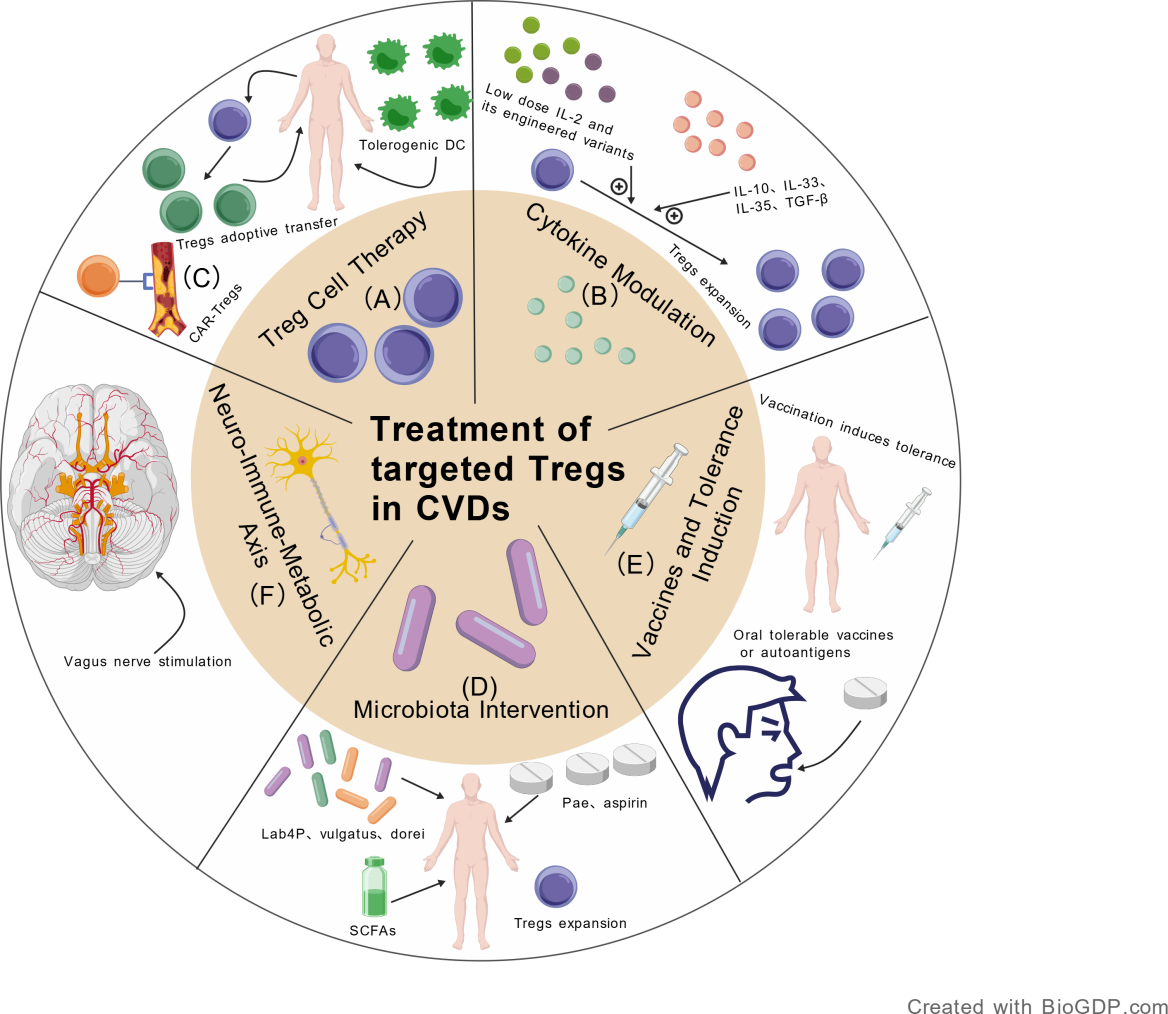
Therapeutic strategies targeting Tregs for CVDs. **(A)** Treg Cell Therapy; **(B)** Cytokine Modulation; **(C)** Targeted Tregs; **(D)** Microbiota Intervention. CVDs, cardiovascular disease; Treg, regulatory T cell. **(E)** Vaccines and tolerance induction **(F)** Neuro-immune-metablic axis

Current cardiovascular Tregs strategies have expanded from simple immunosuppression focused on restoring Tregs numbers and general function to multi-dimensional interventions including cell therapy, cytokine modulation, and metabolic reprogramming. Future directions will focus on enhancing Tregs specificity, stability, and tissue targeting, and exploring therapeutic windows and combination potential across different CVDs stages **Figure 6**.

### 5.1 Treg cell therapy

Treg cell therapy primarily involves ex vivo expansion and reinfusion of autologous or allogeneic Tregs (adoptive transfer), or *in vivo* promotion of Tregs proliferation and functional stability via agents like low-dose IL-2. It aims to rebuild immune tolerance by enhancing or restoring normal Tregs immunosuppressive function to treat chronic inflammatory diseases. This section focuses on Tregs adoptive transfer.

Tregs adoptive transfer involves three steps: isolating autologous or allogeneic Tregs (ensuring purity/function); ex vivo expansion or induced Tregs (iTregs) generation with functional optimization; and infusion of purified cells into patients for targeted therapy (189). Clinically, Tregs adoptive transfer has proven safe and feasible in multiple trials for autoimmune diseases and organ transplantation (86, 190–192).

*In vitro* and *in vivo* studies show transferred functional Tregs can slow progression in many CVDs models, reducing fibrosis and enhancing proliferation of damaged cardiomyocytes without major adverse effects (193). Preliminary clinical data show ex vivo-expanded human Tregs effectively prevent/treat graft-versus-host disease and type 1 diabetes (42), supporting their potential for CVDs. The field is advancing toward precision, with antigen-specific Tregs accurately recognizing pathogenic antigens for enhanced targeting and reduced systemic immune impact (137).

Tregs adoptive transfer effectively inhibits macrophage-like phenotypic conversion, migration, and survival of VSMCs. Early iTregs transfer reduces key pro-inflammatory factors like VSMCs via TGF-β signaling, attenuates plaque burden, and limits atherosclerosis progression. iTregs show superior stability and function to tTregs under inflammatory/high-salt conditions, as macrophage-like VSMCs impair tTregs but not *in vitro* induced iTregs stability (83). Treating naive CD4^+^ T cells with Aza *in vitro* converts them to iTregs via Dnmt1-mediated FOXP3-TSDR demethylation and Foxp3 upregulation. Intravenous infusion of these iTregs increased peripheral Tregs and attenuated atherosclerosis in ApoE-/- mice (189). Intravenous injection of DCs pre-treated with LDL protein ApoB100 and IL-10 reduced effector T cell proliferation, inhibited IFN-γ production, and increased Tregs generation *in vivo*. Splenocytes showed reduced proliferative response to ApoB100 and decreased Th1/Th2 immunity against it. Splenic CD4^+^ T cells suppressed ApoB100-reactive T cell activation in a Tregs-like manner, lowering anti-LDL autoimmunity and reducing atherosclerotic lesion area (194). Adoptive Tregs transfer shifted plaque composition toward a stable phenotype by reducing inflammatory cytokines and MMP-2/9 expression while enhancing P4Hα1 in carotid plaques, lowering plaque rupture incidence in ApoE-/- mice (75). In hypertrophic cardiomyopathy hearts, Tregs increase with disease progression but show altered immunosuppressive phenotype. Lymphocyte depletion worsens adverse remodeling, while Tregs transfer reduces fibrosis, macrophage infiltration, and improves systolic dysfunction (195). In mouse experimental MI models, adoptive Tregs transfer reduced infarct size and improved left ventricular remodeling and function (196).

Furthermore, chimeric antigen receptor (CAR) technology has advanced cell and gene therapy. Applying CARs to Tregs creates CAR-Tregs therapy. VCAM-1-targeting CAR-Tregs effectively homed to aneurysm sites, suppressed local inflammation, reduced aortic dilation, and attenuated abdominal aortic aneurysm progression (197).

Adoptive Treg transfer therapy focuses on isolating a patient’s own Tregs, expanding them *in vitro*, and then reinfusing them. However, this autologous cell therapy faces significant manufacturing challenges. These include limited cell sources, insufficient *in vitro* expansion efficiency, and inconsistent product quality. The emergence of induced pluripotent stem cell (iPSC) technology has been proposed as a potential approach to address the cell source and scalability bottlenecks of traditional Treg cell therapy. Unlike autologous Treg strategies that rely on isolation and expansion from a patient’s peripheral blood, iPSCs possess potential for unlimited self-renewal and multilineage differentiation. Theoretically, they can differentiate into nearly all types of immune cells, including CD4^+^FOXP3^+^ regulatory T cells. This characteristic makes it possible to establish standardized, allogeneic Treg products. It bypasses core manufacturing challenges associated with autologous Tregs, such as limited cell numbers and variable *in vitro* expansion efficiency. This significantly reduces treatment costs and inter-individual variability (198). Furthermore, iPSCs can be combined with gene editing technologies to construct functionally enhanced, antigen-specific Tregs. The differentiated iPSC-Tregs can acquire potent antigen-specific suppressive functions. For example, a recent study confirmed that by inducing iPSC-derived CD4^+^ T cells to highly express FOXP3 and engineering them with a CAR, the resulting iPSC-CAR-Treg-like cells could effectively suppress the proliferation of allogeneic CD8^+^ T cells *in vitro*. They also demonstrated immunosuppressive effects comparable to natural Tregs in a mouse model of xenogeneic graft-versus-host disease (GvHD) (199). This strategy offers new possibilities for precisely targeting immunosuppression to diseased sites and reducing systemic side effects. Although the clinical application of iPSC-Tregs in cardiovascular diseases is still in the early exploratory stage, related basic research and clinical translation are progressing rapidly. Currently, iPSC-derived immune cells, such as CAR-T and CAR-NK cells, have entered early-stage clinical trials in the field of cancer immunotherapy. This has preliminarily validated their safety and feasibility (200). Advances in controlling immunogenicity of iPSC derivatives and developing large-scale differentiation processes compliant with Good Manufacturing Practice are also being pursued (201). Whether iPSC-Tregs will become a viable approach for cardiovascular immunotherapy remains to be determined through further research.

Despite its potential, clinical translation of Treg cell therapy faces multiple challenges. Firstly, insufficient *in vitro* expansion efficiency remains a technical limitation. Tregs constitute only 2%-3% of CD4+ T cells in peripheral blood, making it difficult to obtain sufficient cell numbers for therapy. Furthermore, the purity and functional stability of the expanded cells exhibit inter-individual variability. Additionally, the *in vivo* survival time of infused cells is short, with adoptively transferred Tregs surviving only a few weeks. This limitation may be even more pronounced in human patients, restricting their long-term efficacy (202). Moreover, Tregs may undergo functional impairment and convert to a pro-inflammatory phenotype, losing their original immunosuppressive function. This plasticity implies that within a chronic inflammatory microenvironment, infused Tregs may lose FOXP3 expression and even transform into Th1-like cells. This could paradoxically exacerbate the inflammatory response (203). Regarding targeted delivery, there is currently a lack of effective solutions to ensure precise homing of Tregs to diseased sites. Although CAR-Treg technology can enhance enrichment at lesions by targeting molecules like VCAM-1, its clinical translation also faces challenges. These include the potential for triggering severe inflammatory responses and off-target effects (204). In terms of safety, a state of long-term immunosuppression may increase the risk of infection and the probability of tumorigenesis. This is particularly a concern when using genetically engineered Tregs, for which long-term safety data is still insufficient (205).

In summary, Treg cell therapy has been investigated for its potential in cardiovascular diseases: stabilizing plaques and preventing acute events in atherosclerosis; controlling acute inflammation and limiting injury in cardiomyopathy/MI; and inducing donor tolerance, reducing rejection, and immunosuppressant dependence in heart transplantation. Both adoptive Tregs transfer and CAR-Tregs can potentially restore immune homeostasis. Clinical translation faces challenges: short survival of infused exogenous Tregs (weeks in animals), potential FOXP3 loss and conversion to Th1-like pro-inflammatory phenotypes in diseased microenvironments, low homing efficiency to lesions, lack of standardized expansion protocols, variable patient-specific activity, and potential long-term risks of excessive immunosuppression, infection, or tumorigenesis. Although adoptive Tregs transfer has demonstrated efficacy in numerous murine models of cardiovascular disease, human data remain limited to autoimmune diseases and transplantation settings. No large-scale clinical trials have evaluated Tregs cell therapy for primary CVDs such as atherosclerosis or heart failure. The promising results from animal studies, while mechanistically informative, should not be equated with clinical efficacy. The substantial challenges of ex vivo expansion, *in vivo* persistence, phenotypic stability, and targeted delivery should be systematically addressed before this approach can realize its therapeutic potential. And next-generation therapies must shift from numerical expansion to quality optimization, using genetic engineering to confer targeting, stability, and anti-apoptotic properties. This areas requiring further exploration.

### 5.2 Cytokine modulation

Beyond adoptive therapy, cytokines like low-dose IL-2 can directly expand and activate Tregs *in vivo*, precisely modulating endogenous signaling pathways to reshape immune balance.

Tregs are highly sensitive to low IL-2 levels due to constitutive high-affinity IL-2 receptor (IL-2Rα/β/γc) expression, enabling IL-2 capture to limit Tconv proliferation while enhancing Tregs immunosuppressive phenotype. An intrinsic, receptor-independent signaling sensitivity, potentially from IL-2 signaling deviation from PI3K/Akt/mTOR and Ras/MAPK pathways, allows activation at much lower concentrations than Tconv require, enabling selective Tregs expansion with low-dose IL-2 (206).

Administering low-dose IL-2 or IL-2/anti-IL-2 complexes (IL-2/c) specifically expands Tregs *in vivo*, improving cardiac fibrosis and reducing myocardial macrophage infiltration/activation to support structure and function (195). A clinical trial administering subcutaneous aldesleukin for 5 days in angina patients showed good safety, with most adverse events mild, and dose-dependent Tregs expansion (207). However, current trial patient numbers are small, necessitating larger trials to confirm long-term safety and efficacy.

Natural IL-2 has drawbacks: short half-life requiring frequent dosing; low doses needed to avoid Tconv activation; and off-target proliferation of other IL-2R-expressing cells. This spurred engineered IL-2 development: IgG-(IL-2N88D)_2_ fusion proteins extend half-life, with the N88D mutant favoring sustained Tregs activation (208). Designing IL-2 variants with increased CD25 dependency and enhanced Tregs selectivity, constructed as Fc-fused homodimers (Fc.IL-2 mutants), yields more pronounced and durable Tregs proliferation (209). The pegylated IL-2 molecule NKTR-358, a novel Tregs stimulant, shows lower IL-2 receptor affinity but induces stronger, more cyclical, and potent Tregs expansion without activity loss during treatment (210). An uncleavable IL-2/CD25 fusion protein enables long-lasting, selective Tregs stimulation with more significant expansion (211, 212). Low-dose IL-2 has been shown to safely expand Tregs and reduce vascular inflammation in cardiovascular diseases. However, its therapeutic window is narrow. Furthermore, high-dose IL-2 can lead to severe cardiac toxicity, including the proliferation of effector T cells, myocardial injury, and capillary leak syndrome (213). Although engineered IL-2 aims to enhance selectivity, current clinical evidence for its use in the cardiovascular field remains very limited. In summary, while engineered IL-2 shows promise in overcoming low-dose IL-2 limitations, clinical trials remain few and exploratory, mostly in autoimmune diseases. CVDs efficacy and side-effect assessments are lacking, necessitating further human trials in CVDs.

**Table 2**.

**Table 2:** Beyond IL-2, cytokines like IL-10, IL-33, IL-35, and TGF-β also critically regulate Tregs function and CVDs pathology.

Cytokine	Core functions and CVDs-related mechanisms	Research status and challenges
IL-10	1. Inactivates macrophages/T cells, modulates atherosclerotic plaque progression, exerts anti-inflammatory effects in human lesions. IL-10-deficient mice show increased AS susceptibility (enhanced T cell infiltration, reduced plaque stability); infusion reduces lesions (78); 2. Inhibits IL-6, TNF-α, mTOR, and NF-κB pathways, increases CD4^+^ Tregs numbers, reduces adipose tissue inflammation, improves metabolic dysregulation, and lowers mean arterial pressure in rats (247, 248).	1. Systematic research in CVDs lags; molecular mechanisms and Tregs regulation are unclear. 2. Targeted delivery systems and large RCTs are needed to validate therapeutic value in CVDs. 3. Inconsistent clinical trial results; high IL-10 levels can be pathogenic in diseases like SLE (249).
IL-33	Reverses visceral adipose tissue inflammation and insulin resistance. In lean mice, IL-10-high Tregs in visceral fat are mostly ST2^+^; this proportion decreases in obesity. IL-33 treatment reverses this decline and maintains ST2^+^ Tregs numbers (250).	Overall research is limited, particularly regarding targeted mechanisms and efficacy in CVDs.
IL-35	1. Inhibits Th1/Th17 cells and DCs, promotes Tregs proliferation/suppressive function, induces iTr35 cells, upregulates IL-10, downregulates IL-17 (79); 2. Binds pulmonary vascular endothelial GP130 receptor, phosphorylates STAT1, reverses pulmonary vascular remodeling in pulmonary hypertension (251); 3. Inhibits DC maturation, modulates Tregs/Th17 balance, prolongs cardiac allograft survival (252); 4. Modulates IFN-γ, PD-1, etc., suppresses CD8+ T cell cytotoxicity, enhances Tregs response, alleviates Kawasaki disease inflammation (253).	A potential target for various CVDs with relatively clear mechanisms.
TGF-β	Exerts anti-atherosclerotic effects; inhibiting its signaling (via neutralizing antibodies) accelerates lesion progression (increased inflammation, reduced collagen). TCR/IL-2 co-stimulation in vitro induces iTregs with high Nrp-1 expression (regulated by SP1). Nrp-1^+^ iTregs are more stable, resist conversion to Th1/Th17 under inflammation, and exhibit stronger T cell proliferation suppression (254).	1. Lacks in vivo protein-level validation of key molecules like Nrp-1. 2. Few clinical translation cases in CVDs; direct in vivo therapeutic effects unclear. 3. Limited recent research progress; requires expanded preclinical and clinical trials.

Endogenous cytokine modulation of Tregs is a promising CVDs strategy but faces challenges like short half-lives, poor targeting, off-target effects, and predominantly preclinical status. Therefore, more durable and specific immune tolerance approaches are needed. Vaccine- or antibody-mediated active/passive immunization represents another key avenue for reshaping immune balance and modifying disease.

### 5.3 Vaccines and tolerance induction

Active immunization with antigens like LDL or apoB-derived peptides attenuates atherosclerosis in apoe^-^/^-^ and ldlr^-^/^-^ mice, with protection relying on specific antibody generation, suppression of pro-inflammatory immune responses, and Tregs activation. Adjuvants are widely used in these studies to enhance immune responses by activating innate immunity (214). Aluminum adjuvants, with low pro-inflammatory activity and strong antibody induction, are widely used in human vaccines (215). Mucosal immunization is an effective strategy for inducing protective immunity. Oral, intranasal, and other mucosal routes can activate regulatory immunity, inducing Tregs expansion and antibody production (216).

Vaccine-based active immunization enhances Tregs-mediated suppression in diseases like AS by modulating autoimmune responses. For example, influenza vaccine lowers MI and stroke risk (217); S. pneumoniae vaccine induces anti-oxLDL cross-reactive antibodies (80, 218); anti-PCSK9 vaccine lowers lipids cost-effectively (219); the anti-oxLDL vaccine MLDL1278A inhibited ox-LDL but showed no clear anti-inflammatory advantage (220).

For passive immunization, Evolocumab (anti-PCSK9 mAb) lowers LDL-C and cardiovascular event risk with good safety (221); Canakinumab (anti-IL-1β mAb) reduces inflammatory markers dose-dependently, with 150mg reducing non-fatal MI and other events without lipid-related adverse effects (222). This confirms “anti-IL-1β anti-inflammatory therapy” works independently of lipid-lowering, offering a new thought for intervening in inflammation-related cardiovascular risk.

Ardehali H’s team proposed an innovative strategy: “tolerogenic vaccines targeting cardiac autoantigens to induce peripheral tolerance.” Oral tolerogenic vaccines against cardiac autoantigens induce Tregs and prophylactically attenuate pressure overload-induced heart failure, confirming an autoimmune-like pathogenesis in HF (223).

Despite promise, clinical translation faces challenges: From a safety perspective, targeting autoantigens carries the inherent risk of triggering autoimmunity, while aluminum adjuvants exhibit inadequate ability to discriminate between different types of immune responses (224). Efficacy seen in murine models often does not translate to human clinical settings. Marked interindividual immune heterogeneity and the lack of reliable Tregs activation biomarkers are two key factors that hinder clinical implementation. Trial costs are substantial. The therapy has an unclear competitive edge over established effective therapies. These two issues further impede its widespread adoption (225).

Vaccine and antibody strategies represent a precision direction for cardiovascular immunotherapy, while gut microbiota indirectly modulates immune responses via metabolites and the microbiota-immune axis, offering a potential indirect pathway.

### 5.4 Microbiota intervention

Gut microbiota plays a key role in CVDs via metabolites, composition, and host immune interactions, making targeted modulation a potential therapeutic strategy.

Short-chain fatty acids (SCFAs), particularly propionate and butyrate from microbial fermentation of dietary fiber, significantly increase Tregs numbers in the colonic lamina propria, maintaining immune tolerance. Specifically, propionate protects against abdominal aortic aneurysm in a Tregs-dependent manner, and significantly attenuates cardiac hypertrophy, fibrosis, vascular dysfunction, and hypertension, suppresses systemic/cardiac inflammation, reduces susceptibility to ventricular arrhythmias, and decreases aortic lesion area (226, 227).

Beyond direct metabolite supplementation, modulating microbiota structure/function with specific agents can indirectly yield cardiovascular protection. For example, paeonol (Pae) remodels gut microbiota, promotes SCFA production, selectively upregulates splenic Tregs frequency, downregulates Th17 proportion, improves Tregs/Th17 balance, and limits atherosclerosis development and collagen deposition (228). Aspirin alters gut microbial composition (e.g., increases Bacteroidetes, lowers Firmicutes/Bacteroidetes ratio), elevates SCFA levels, optimizes bile acid profile, and enhances anti-inflammatory/immunomodulatory effects by rebalancing Tregs/Th17 ratio and upregulating CD39/CD73 adenosine signaling (229).

Direct supplementation of beneficial microbes (probiotics) or microbiota transplantation provides more direct evidence for intervention. In animal studies, Lab4P supplementation increased plasma HDL, lowered LDL/VLDL, reduced hepatic pathogenic gene expression, decreased bone marrow macrophage/T cell frequency, and modulated lipids to reduce plaque burden and enhance stability (230). Clinical observation found reduced abundance of Bacteroides vulgatus and Bacteroides dorei in CAD patients. Gavage with these live Bacteroides species reduced LPS production, suppressed pro-inflammatory immune responses, and effectively attenuated atherosclerotic lesions (231).

However, currently, human clinical trials have not yet consistently demonstrated that modulating the gut microbiota can significantly reverse cardiovascular outcomes. Most of the evidence remains at the level of mechanistic exploration. Clinical translation faces challenges: evidence largely from animal studies with incomplete mechanisms; SCFA targets and *in vivo* dynamics unclear; high human microbiota heterogeneity limits animal-to-human prediction; biomarkers to identify responders are lacking; and long-term safety, colonization stability, and personalized protocols for microbiota transplantation require resolution (232). Furthermore, genetic variations in short-chain fatty acid receptors can completely negate the cardiovascular protective effects of dietary fiber. This indicates that the impact of individual genetic background on therapeutic efficacy cannot be overlooked. These factors further limit the clinical translation of this strategy (233).

### 5.5 The neuro-immune-metabolic axis

The vagus nerve transmits peripheral pro-inflammatory signals to the brain (due to afferent fiber sensitivity to interleukins and prostaglandins), activating the HPA axis to release cortisol from the adrenal cortex. Efferent vagal anti-inflammatory effects, mediated via vagovagal reflexes, inhibit macrophage cytokine release like TNF-α, making vagus nerve stimulation a potential therapy for autoimmune and cardiovascular diseases (234). Studies show the vagus nerve modulates inflammation by regulating CD4^+^ T cell differentiation. Right cervical vagotomy worsened viral myocarditis by inhibiting the cholinergic anti-inflammatory pathway, increasing splenic Th1/Th17 and decreasing Th2/Tregs proportions, promoting CD4^+^ T cell differentiation, aggravating myocardial injury/cell infiltration, and impairing cardiac function—effects reversed by the α7nAChR agonist PNU282987 (235). Chronic psychosocial stress activates the SAM and HPA axes, chronically elevating heart rate/blood pressure, promoting visceral fat accumulation, inducing chronic inflammation to accelerate atherosclerosis, and altering gut microbiota/barrier function. Perinatal/infant stress can epigenetically modify SAM function long-term, increasing adult susceptibility to hypertension and other CVDs pathologies.(Stress and Metabolic Disease - Sociality, Hierarchy, Health: Comparative Biodemography - NCBI Bookshelf).The stress response system also activates CRH, AVP, glucocorticoids, and catecholamines. Stress hormones have dual immune effects: systemically suppressing Th1/pro-inflammatory responses and inducing Th2 bias to prevent excessive inflammation; locally potentially promoting pro-inflammatory responses. Dysfunctional stress systems (hyper- or hypoactive) are linked to abnormal systemic anti-inflammatory feedback and local pro-inflammatory factor overactivity, potentially contributing to chronic inflammation and immune-related diseases (236). Thus, modulating the neuro-immune-metabolic axis and Tregs via vagus nerve stimulation and autonomic regulation is a novel CVDs therapeutic direction (237).

Metabolically, Tregs’ low glucose uptake and reliance on oxidative phosphorylation/fatty acid oxidation make targeting PI3K/Akt/mTOR and AMPK pathways promising for CVDs.

Vagus nerve stimulation (VNS) has entered the early stages of clinical investigation for cardiovascular diseases. It has been preliminarily confirmed that treating cardiovascular diseases with VNS is safe and feasible. The field still faces multiple challenges: severe lack of clinical trials and translation, mostly theoretical; key scientific questions remain, e.g., precise neural control mechanisms for specific T cell subset differentiation, bidirectional immune effects of stress hormones, and how to specifically intervene in T cell metabolism without systemic sympathetic/parasympathetic side effects; current neuromodulation techniques (e.g., VNS) are invasive and non-specific; individual heterogeneity hinders universal therapy development. Future work requires interdisciplinary efforts to dissect interaction networks and develop non-invasive, precise modulation techniques and personalized strategies.

### 5.6 Other therapies

Myocardial infarction enhances splenic extramedullary hematopoiesis (EMH), and its absence exacerbates post-MI tissue injury. PGI2 receptor-deficient mice show enhanced splenic EMH and improved post-MI cardiac recovery (238). In the pathological progression from heart failure to ischemic HF, MI, and post-ischemic repair, local inflammation after ischemic injury hinders repair, creating a lack of therapies that significantly improve cardiac structure/function. Cortical bone stem cell-derived exosomes, rich in miR-182/183, mediate macrophage reparative phenotype polarization and metabolic reprogramming (increased oxidative phosphorylation, reduced ROS) via the RASA1 axis, improving cardiac function (239). Anti-inflammatory therapies like targeted drugs, mesenchymal stem cells, and colchicine have achieved breakthroughs, with research on new targets like CXCR4 and CD47 advancing (240). Allogeneic adipose-derived stromal cell (ASC) therapy shows significant efficacy in ischemic heart failure without complications or serious adverse events, though it may not benefit chronic, unselected patients (241–244). Colchicine significantly reduces cardiovascular event risk (e.g., cardiovascular death, spontaneous MI) in patients with chronic coronary disease (245, 246).

Based on current understanding of Tregs in CVDs, various interventions aimed at enhancing Tregs number or function have been developed. Key considerations for advancing these approaches include ensuring precise homing and persistence of adoptive Tregs, preventing functional exhaustion and phenotypic conversion, avoiding excessive immunosuppression, and developing personalized strategies. Addressing these issues will be important for determining the clinical utility of next-generation therapies.

## 6 Modulating factors

This section comprehensively characterizes a panel of endogenous and exogenous factors that potently modulate the differentiation, functional stability, and immunosuppressive capacity of Tregs. This complements the active Treg-targeted interventional approaches addressed earlier in the review. As summarized in **Table 3** of the manuscript, these factors include endogenous sex hormones, commonly prescribed clinical medications, and inflammatory microenvironmental regulators. All of them offer opportunities for drug repurposing and the development of combinatorial Treg-modulating therapies (**Table 3**).

**Table 3:** Beyond the active interventions above, many endogenous hormones and common clinical drugs significantly modulate Tregs, offering opportunities for drug repurposing and combination therapy.

Factor	Primary mechanism of action	Effect on Treg cells
Estrogen	Estradiol (E2) binds ERα in Tregs, forming a complex that interacts with FOXP3 protein, occupies the FOXP3 promoter and conserved non-coding elements, activates the promoter, and sustains FOXP3 expression to maintain Tregs function. ERα antagonism blocks this, leading to FOXP3 loss and impaired Tregs function (255).	Physiologically, rising serum estradiol in the late follicular phase correlates with increased peripheral Tregs in premenopausal women, while postmenopausal or recurrent miscarriage women show reduced Tregs number/function (256). In EAE models, estrogen therapy expands Tregs and upregulates PD-1 via ERα, enhancing suppression (257, 258). However, in IBD, estrogen has dual roles, mediating immunosuppressive or immunostimulatory effects via different cell types (259–261).
Progesterone	Progesterone enhances IL-2/STAT5 signaling while inhibiting IL-6-mediated STAT3 activation, promoting naive T cell differentiation toward Tregs over Th17 (262).	Progesterone increases Tregs proportion and IL-10 expression, enhancing their suppressive function. It converts CD4^+^CD25^-^ T cells into CD4^+^CD25^+^ Tregs, contributing to maternal-fetal immune tolerance during mid-gestation (263).
Androgen	Acts via functional androgen response elements (AREs) within the FOXP3 locus.	Androgens regulate T cells only in ovulating women during the menstrual cycle, not significantly in men: binding to the androgen receptor in T cells targets AREs in the Foxp3 locus, altering histone H4 acetylation without affecting specific CpG methylation, thus epigenetically upregulating Foxp3 expression to promote Tregs differentiation and increase Tregs levels (264).
Metformin	Activates AMPK signaling (upregulates p-AMPK), inhibits mTOR signaling (downregulates p-mTOR), directly suppresses p-STAT3 expression, upregulates the key Tregs transcription factor Foxp3, and downregulates pro-inflammatory IL-17 (265).	Metformin reduces Th17 and increases Treg cells percentages and related cytokine levels (266).
Statins	1. Inhibit HMG-CoA reductase, reducing mevalonate production and blocking geranylgeranylation. This promotes CpG demethylation in the Foxp3 proximal promoter to induce Foxp3 expression and reduces Smad6/7, relieving their blockade of TGF-β signaling. 2. Inhibit phosphorylation of PI3K-Akt-mTOR and ERK pathways.	Simvastatin induces Tregs generation in the inflammatory microenvironment of atherosclerotic plaques. Statins promote Tregs generation from primary T cells and enhance function of existing Tregs (267, 268).
Vitamin D	Its active form, 1,25(OH)2D3, binds the vitamin D receptor (VDR); the complex then binds vitamin D response elements (VDREs).	Inhibits pro-inflammatory cytokine production (IFN-γ, IL-17, IL-21), stimulates high CTLA-4 and FoxP3 expression, suppresses proliferation of normal reactive T cells, inhibits Th1 and promotes Th2 differentiation, and promotes differentiation of resting CD4^+^CD25^-^ T cells into a FOXP3+ regulatory lineage (269).
TNF-α Inhibitors	Bind membrane TNF on monocytes, enhancing monocyte TNF expression and promoting its binding to TNF-RII on Tregs, thereby expanding functional FOXP3^+^ Tregs via an IL-2/STAT5-dependent mechanism and inhibiting Th17 cells.	Anti-TNF therapy significantly reverses subclinical cardiovascular dysfunction in RA, AS, and PsA, reduces myocardial inflammation, lowers serum inflammatory markers (CRP, ESR) and disease activity, with a trend toward improved myocardial perfusion (270). It modulates activation and generation of Th1, Th17, and Treg cells, reducing CVDs risk (271).
Aspirin	Promotes IL-11 transcription via the transcription factor CREB.	Aspirin increases FOXP3 and IL-4 content in T cells, inhibiting naive T cell differentiation into Th17 and Th1 cells (272).
Inflammatory Environment	The transcriptional regulator Blimp1 negatively regulates IL-6/STAT3-mediated Dnmt3a expression/activity, inhibiting methylation of the FOXP3 CNS2 region, thereby maintaining stable Foxp3 expression and Tregs function.	In severe inflammatory microenvironments, Tregs identity and immunosuppressive function are vulnerable. Blimp1 effectively prevents downregulation of FOXP3 protein expression and inhibits trTregs from abnormally acquiring "effector functions" (136).

## 7 Discussion

The recognition of Tregs research through the 2025 Nobel Prize in Physiology or Medicine underscores the fundamental importance of effector-regulatory immune balance in maintaining self-tolerance. This review has examined the role of Tregs in CVDs, a non-classical autoimmune disease: they act as tissue repairers and metabolic regulators but can also drive pathology in specific contexts. This functional dysregulation involves not only reduced immunosuppressive capacity but also plastic conversion towards pro-inflammatory phenotypes. This understanding suggests that future diagnostic and efficacy assessment approaches may need to extend beyond quantifying Tregs numbers to include analysis of their functional status, epigenetic profiles, and metabolic activity.

The clinical translation of Tregs therapies faces several challenges. First, heterogeneity and plasticity. Tregs are not homogeneous; their phenotype and function differ markedly across tissues like the arterial wall and heart. In the chronic inflammatory milieu of advanced CVDs, they easily lose FOXP3 and convert to Th1/Th17-like pro-inflammatory phenotypes. Their tissue-specific adaptation and metabolic checkpoints (nutrient availability, mitochondrial function) critically influence local inflammation regulation. Second, precision and safety concerns. Achieving specific Tregs enrichment at disease sites is essential to minimize risks of infection and tumorigenesis from systemic immunosuppression. CAR-Tregs and nanocarrier-targeted delivery are under investigation to address this. Third, treatment timing and individual variability. The inflammatory landscape dynamically changes across CVDs stages, necessitating identification of optimal intervention windows. Patient age, sex, comorbidities, and gut microbiota all affect efficacy, requiring treatments that are both personalized and dynamically adjustable. Fourth, the clinical translation gap. Findings from animal studies (genetically identical, controlled environment) are difficult to directly translate to immunologically heterogeneous human populations with multiple comorbidities and long-term medication use.

Current strategies to enhance Tregs number or function (cell therapy, cytokine modulation, vaccine/microbiota intervention) show promise in animal models and early clinical studies. Integration of neuro-immune circuits also opens new paths for regulating Tregs activity. However, challenges persist regarding human Tregs heterogeneity, pro-inflammatory microenvironment instability, and off-target effects. A recurring theme throughout this review is the gap between mechanistic insights gained from preclinical models and their translation to human disease. First, species differences in immune cell subsets, receptor expression, and cytokine signaling pathways can fundamentally alter therapeutic responses. As mentioned above, the markedly higher cytotoxicity of human CD57^+^CD8^+^ T cells compared to murine counterparts and the diminished efficacy of Tregs adoptive transfer in human studies relative to mouse models exemplify these differences. Second, the controlled environment of animal studies including genetically identical subjects, standardized diets, absence of comorbidities stands in stark contrast to the heterogeneous human population with variable genetic backgrounds, polypharmacy, and multiple chronic conditions. Third, the time course of disease differs substantially: atherosclerotic plaques develop over decades in humans versus weeks to months in mice, with corresponding differences in plaque composition, fibrosis, and immune cell kinetics.

Findings derived solely from animal models should be viewed as hypothesis-generating rather than clinically established. Where human data exist, they often provide correlative rather than causal evidence, identifying associations between Tregs subsets and disease states without establishing therapeutic efficacy. The few instances of cross-species validation such as miR-155 in myocarditis provide encouraging proof-of-concept but remain exceptions rather than the rule.

Future research should prioritize five directions: 1) Using single-cell multi-omics and spatial transcriptomics to decipher Tregs heterogeneity and dynamic maps across CVDs stages and tissues, identifying protective and pathogenic subsets. 2) Integrating AI to build clinical prediction models, identify efficacy/prognostic biomarkers, and discover new targets. 3) Developing precision combination therapies, such as pairing Tregs therapy with lipid-/blood pressure-lowering drugs to reshape the immune microenvironment. 4) Identifying transcriptional and metabolic switches that maintain Tregs phenotype stability for drug development targeting. 5) Advancing tool development like antigen-specific CAR-Tregs and targeted nanodrug delivery systems to reduce systemic immunosuppression risks.

In summary, Tregs-targeted immunotherapy represents an actively investigated approach for CVDs prevention and treatment. The core lies not in pursuing Tregs quantity but in understanding how disease signals and the microenvironment influence their functional subsets. Continued investigation of Tregs biology and systematic efforts to address clinical translation challenges may ultimately enable the development of therapies that can move beyond delaying disease progression toward restoring immune homeostasis.

## 8 Conclusion

Tregs are central coordinators of peripheral immune tolerance, playing an indispensable role in suppressing cardiovascular inflammation and promoting tissue repair. The foundational work recognized by the Nobel Prize provides a solid theoretical basis for Tregs-targeted therapies. However, as this review elucidates, the heterogeneity, plasticity, and tissue-specificity of Tregs function demand more refined and precise therapeutic strategies than before. Existing strategies—cell therapy, cytokine modulation, vaccines, and microbiota intervention—show great potential in preclinical and early clinical research, yet their safety, efficacy, and long-term stability require rigorous validation in larger-scale clinical trials. Future success will inevitably depend on a multidimensional perspective integrating systems immunology, precision medicine, and cutting-edge biotechnology. Only through this integration can the vast potential of Tregs in cardiovascular disease prevention and treatment be fully unlocked, achieving the ultimate goal of transitioning from disease management to health restoration.
